# Metabolic activation of LDHA by ERRα represses inflammasome-dependent pyroptosis and promotes ovarian cancer chemoresistance

**DOI:** 10.1038/s41419-026-08986-6

**Published:** 2026-06-17

**Authors:** Yuan Ren, Jingxuan Ye, Yonghong Zhang, Guanyu Ruan, Yashi Shi, Jincheng Ma, Hao Lin, Liang Wang, Liying Wang, Xite Lin, Maotong Zhang, Nan Wang, Xiaodan Mao, Pengming Sun

**Affiliations:** 1 Fujian Clinical Research Center for Gynecological Oncology, Fujian Maternity and Child Health Hospital, College of Clinical Medicine for Obstetrics & Gynecology and Pediatrics, Fujian Medical University, Fuzhou, Fujian China; 2 Fujian Key Laboratory of Women and Children’s Critical Diseases Research, Fujian Maternity and Child Health Hospital, Fuzhou, Fujian China; 3 Gynecology Department, Jinjiang Municipal Hospital, Jinjiang, Fujian China; 4 Qingdao Hospital, University of Health and Rehabilitation Sciences (Qingdao Municipal Hospital), Qingdao, Shandong China; 5 Department of Gynecology, Fujian Maternity and Child Health Hospital, College of Clinical Medicine for Obstetrics & Gynecology and Pediatrics, Fujian Medical University, Fuzhou, Fujian China; 6 School of Group Medicine and Public Health, Peking Union Medical College, Beijing, China

**Keywords:** Cancer metabolism, Ovarian cancer

## Abstract

Chemoresistance remains a major unmet challenge in the clinical management of ovarian cancer. As a metabolism-associated malignancy, the poor prognosis and frequent chemoresistance of ovarian cancer are closely linked to metabolic reprogramming. In this study, we identify estrogen-related receptor α (ERRα) as a key regulator of metabolic plasticity and chemoresistance in ovarian cancer. Bioinformatic analyses of pan-cancer datasets and chemoresistant ovarian cancer samples reveal that high expression of ERRα and the glycolytic rate-limiting enzyme lactate dehydrogenase A (LDHA) is associated with poor clinical outcomes. Elevated serum LDH levels in chemoresistant patients further underscore the importance of metabolic reprogramming in the development of chemoresistance. Mechanistically, ChIP-seq, dual-luciferase reporter assays, and enzymatic colorimetric assays demonstrate that ERRα directly binds to the LDHA promoter region (5′-AGAAGGTCG-3′), activating its transcription and enhancing glycolysis and the production of its end-product, lactate. Scanning electron microscopy, immunofluorescence, Western Blot, and other molecular functional assays show that the ERRα/LDHA axis drives lactate accumulation, downregulates inflammasome-related proteins (NLRP3, caspase-1, GSDMD and GSDMD-N), thereby suppressing pyroptosis and promoting resistance to cisplatin, carboplatin, and paclitaxel in ovarian cancer cells. Pharmacological inhibition of ERRα with XCT790 restores sensitivity to chemotherapeutic agents. In a mouse xenograft model, targeting ERRα enhances the therapeutic efficacy of chemotherapy. Collectively, these findings reveal that the ERRα–LDHA axis increases lactate production, bridging glycolytic metabolism and the suppression of pyroptosis, thereby facilitating chemoresistance in ovarian cancer. Targeting the ERRα/LDHA pathway and developing ERRα inhibitors may represent promising strategies to overcome chemoresistance in ovarian cancer.

**Figure:**
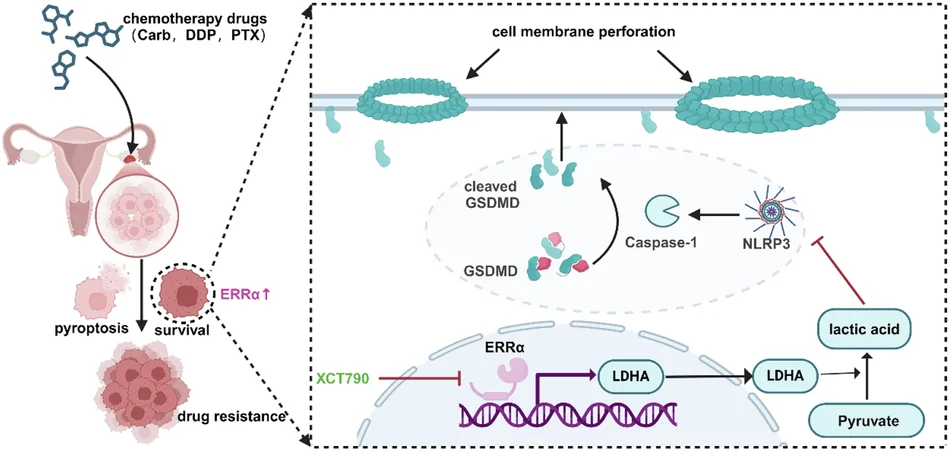
ERRα targets the promoter region of LDHA, promoting its transcription and the production of the glycolytic product lactate, inhibiting the NLRP3/caspase-1/GSDMD pathway, reducing cell pyroptosis, and helping ovarian cancer cells resist the cytotoxic effects of carboplatin and paclitaxel. Created in BioRender. Ren, Y. (2025) https://BioRender.com/qeh0dw8.

ERRα targets the promoter region of LDHA, promoting its transcription and the production of the glycolytic product lactate, inhibiting the NLRP3/caspase-1/GSDMD pathway, reducing cell pyroptosis, and helping ovarian cancer cells resist the cytotoxic effects of carboplatin and paclitaxel. Created in BioRender. Ren, Y. (2025) https://BioRender.com/qeh0dw8.

## Introduction

According to 2022 global cancer statistics, ovarian cancer (OC) ranks third in incidence and second in mortality among female reproductive system cancers, with 206,839 deaths in 2022 alone [1]. The high mortality rate is primarily due to late-stage diagnosis, peritoneal metastasis, and chemotherapy resistance [2]. Approximately 75% of patients who initially respond to treatment relapse within two years and become resistant to available chemotherapeutic drugs [3, 4]. Classic chemotherapeutic agents, such as platinum-based drugs and paclitaxel (PTX), inhibit DNA synthesis and cell proliferation, but resistant cells exhibit enhanced DNA repair mechanisms [5]. Key factors contributing to OC chemoresistance include altered drug metabolism, metabolic dysregulation, alterations in drug targets, inhibition of cell death, and impaired DNA damage repair [6]. In particular, chemoresistance is closely associated with the metabolic characteristics of tumors, especially in advanced stages [7]. As a result, research has increasingly focused on the role of cellular metabolism in tumor chemoresistance.

Tumor cells preferentially undergo aerobic glycolysis instead of oxidative phosphorylation (OXPHOS), a phenomenon first described by Warburg. Despite a lower ATP yield, glycolysis efficiently provides biosynthetic intermediates, such as NADPH, to sustain rapid growth [8]. Recent studies have shown that the dysregulation of glycolytic enzymes and transport proteins, including GLUT1, HK, PKM2, and LDHA, is linked to chemotherapy resistance [9]. Glycolysis promotes doxorubicin resistance in osteosarcoma cells by increasing glucose consumption and lactate production, thereby facilitating ATP production [10]. LDHA, a key enzyme in glycolysis, catalyzes the conversion of pyruvate to lactate and is critical for cancer cell proliferation, stemness, and metastasis [11, 12]. Furthermore, the acidic environment generated by lactate contributes to chemotherapy resistance [2].

Estrogen-related receptor α (ERRα/ESRRA) is a transcription factor primarily located in the nucleus and plays a significant role in the initiation and progression of OC. Recent studies have shown that ERRα is highly expressed in the tumor tissues of patients with OC [13], and its expression increases as the tumor progresses in both pathological grading [14] and clinical staging [15]. Previous studies have established a negative correlation between ERRα expression and overall survival (OS) [16], although its association with progression-free survival (PFS) remains controversial [13, 16]. Additionally, ERRα has been shown to promote the expression of vascular endothelial growth factor (VEGF) in OC cells, facilitating angiogenesis and OC progression [17]. Therefore, ERRα amplification is crucial for the malignant transformation of ovarian cells and is a key molecular event in OC development, making it a potential target for early screening and prognostic assessment. ERRα is also central to cellular energy metabolism. We have previously demonstrated that ERRα regulates glycolytic reprogramming to resist cisplatin (DDP)-induced pyroptosis in endometrial cancer (EC), contributing to cancer chemoresistance [18]. However, the exact mechanism by which glycolysis inhibits pyroptosis in OC requires further investigation. We hypothesized that ERRα-regulated metabolic reprogramming may also help OC cells resist PTX- or PTX-induced pyroptosis, with lactate, a product of glycolysis, acting as a key intermediate substance mediating this process. Exploring the underlying mechanisms of chemotherapy resistance in OC may help identify novel genes that serve as biomarkers for platinum or PTX sensitivity, and facilitate the development of targeted therapies to enhance chemotherapy efficacy, improve prognosis, and reduce recurrence and mortality in OC patients.

## Methods and materials

### Bioinformatics data analysis

Transcription and prognosis data for ESRRA and LDHA were obtained from the Gene Expression Profiling Interactive Analysis (GEPIA) database (http://gepia.cancer-pku.cn/index.html). For pan-cancer prognosis evaluation, the cut-off value for LDHA and ESRRA was set at 50%. In OC, the OS cut-off value for ESRRA was determined based on quartiles. Relevant data for OC patients or OC cell lines, including GSE159791, GSE73935, GSE15372, and GSE1926, were retrieved from the Gene Expression Omnibus (GEO) database (https://www.ncbi.nlm.nih.gov/gds/?term=). Gene expression data from these datasets were extracted and processed for analysis, comparing relative mRNA levels. The STRING database (https://cn.string-db.org/) was used to predict the interaction between ESRRA and LDHA. The UCSC Genome Browser (https://genome.ucsc.edu/) and the JASPAR (RRID: SCR_003030) database were used to predict transcription factors upstream of LDHA. Sequencing data were analyzed using the Gene Ontology (GO) database (https://www.geneontology.org/), which classifies gene functions into three major categories: biological process (BP), cellular component (CC), and molecular function (MF).

### Clinical data analysis

Clinical samples were retrospectively collected from OC patients diagnosed between 2005 and 2023 at Fujian Maternity and Child Health Hospital. Patients were eligible for inclusion if complete clinicopathological data were available. Patients were excluded if they had a history of other malignancies or had received immunotherapy or endocrine therapy within 6 months before diagnosis. These inclusion and exclusion criteria were predefined before data collection and analysis. No blinding was performed during the experiment or outcome assessment. A total of 124 patients met the inclusion criteria and were included in the study. Among them, 105 patients had available chemosensitivity data and were further analyzed for drug response–related investigations. All participants signed informed consent forms, and clinical data were collected for further analysis. Tumor tissues were obtained during surgery, digested with collagenase to prepare single-cell suspensions, and the CCK-8 assay was employed to assess the inhibitory effects of different concentration combinations of DDP + PTX and carboplatin (Carb) + PTX on tumor cells. The IC₅₀ values were calculated using the four-parameter logistic equation. This study was approved by the Ethics Committee of Fujian Maternity and Child Health Hospital (Approval No.: 2022KYLLR03044).

### Cleavage under targets and tagmentation (CUT &Tag)

CUT&Tag was performed using a commercial kit (N259-YH01, Novoprotein, Jiangsu, China). Briefly, cells were immobilized on concanavalin A-coated magnetic beads and permeabilized with digitonin. Antibody-guided pA-Tn5 transposase was recruited to target DNA sites, followed by adaptor ligation and PCR amplification to generate sequencing-ready libraries. Libraries were purified with AMPure beads and quality-checked using an Agilent Bioanalyzer 2100. Sequencing reads were mapped to the reference genome, and peaks were annotated using ChIPseeker to identify associated genes and genomic regions.

### Dual-luciferase reporter gene

The JASPAR database identifies potential binding sites of ERRα in the LDHA promoter region. The LDHA promoter sequence (NC_000011.10:18392563-18394563) corresponding to the transcription start site was amplified by PCR and inserted into the pGL3-basic plasmid. Three progressively shorter LDHA promoter fragments were ligated to the pGL3-basic plasmid: LDHA-p1 (18393533 to 18394563), LDHA-p2 (18393913 to 18394563), and LDHA-p3 (18394163 to 18394563). The potential sequence 5’-AGAAGGTCG-3’ was mutated and named mut-LDHA-p1 -p1. In a 24-well plate containing Lipofectamine 3000 (L3000075, Invitrogen, USA), 293T cells were co-transfected with the ERRα transcription factor, the luciferase reporter gene-marked LDHA promoter, and sea cucumber luciferase. After 48 h, the firefly and sea cucumber luciferase activities were measured using the Dual-Lite luciferase assay system (DD1205, Vazyme, Nanjing, China) with a microplate reader (Gen 1), and the ratio of firefly to sea cucumber luciferase activity was calculated.

### Cell lines and cell culture

Human 293T cells (Fujian Provincial Maternity and Child Health Hospital Gynecological Oncology Laboratory, Fujian, China, RRID: CVCL_0063), human SKOV3 (RRID: CVCL_0532) and ES2 (RRID: CVCL_3509) OC cells (Fenghui Biotechnology Co., Ltd., Hunan, China) were cultured in DMEM (11965092, Gibco™, New York, USA), McCoy’s 5 A (16600082, Gibco™, New York, USA), and PRMI-1640 (11875119, Gibco™, New York, USA) media, all containing 1% penicillin-streptomycin (15140122, Gibco™, New York, USA) and 10% fetal bovine serum (10091148, Gibco™, New York, USA), in a humid environment at 37 °C (5% CO_2_, 95% air). All cell lines were validated by short tandem repeat (STR) DNA profiling analysis and were confirmed to be free of mycoplasma contamination before use. The lentiviral vector LV-sh-ESRRA (named siERRα) was synthesized by Genechem (Shanghai, China). The siRNA target sequence in ERRα (LV-ESRRA-RNAi, 55,367–1) is 5’-TAC CAC TAT GGT GTG GCA T-3’. The lentiviral vector used for overexpressing ERRα was named ovERRα. The negative control was purchased from Genechem (Shanghai, China). We silenced ERRα expression using a specific shRNA lentiviral vector (siERRα) and its empty vector (as a control), and overexpressed the ERRα vector and its empty vector (as an overexpression control), transfecting them into stable clones marked with EGFP. ERRα was overexpressed in SKOV3 and ES2 cells, named SKOV3^-ovERRα^ and ES2^-ovERRα^, respectively. Additionally, the SKOV3 and ES2 cells with downregulated ERRα were named SKOV3^-siERRα^ and ES2^-siERRα^.

### Western blotting (WB)

Standard techniques were used for protein quantification, separation, transfer, and blotting in SKOV3 and ES2 cells. Primary antibodies used for immunoblotting included: ERRα (1:500, ab76228, Abcam, London, UK, RRID: AB_1523580), ERRα (1:500, E1G1J, Cell Signaling Technology, Massachusetts, USA, RRID: AB_2750873), LDHA (1:1000, A1146, Wuhan, China, RRID: AB_2758572), NLRP 3 (1:500; 19,771-1-AP, Proteintech, Wuhan, China, RRID: AB_10646484), caspase1 (22915-1-AP, Proteintech, Wuhan, China, RRID: AB_2876874), GSDMD (1:500, E9S1X, Cell Signaling Technology, Massachusetts, USA, RRID: AB_2921367), GSDMD-N (1:1000, ab215203, Abcam, London, UK, RRID: AB_2916166), β-actin (1:2000, YM 3028, Immunoway, USA, RRID: AB_2629465).

### RT-qPCR

Cells in the culture dish were digested with trypsin, and the cell pellet was collected by centrifugation, followed by the addition of RNA Extraction to lyse the cells. The cell lysate was thoroughly vortexed. Samples were collected, and the supernatant was transferred to a new tube. For every 1 ml of RNA extract, 0.25 ml of chloroform was added. After centrifugation, the upper aqueous phase was transferred to a new tube. RNA was denatured by mixing it with isopropanol. RNA was washed with 75% ethanol. First-strand cDNA synthesis and preparation of the PCR master mix (A2791, Promega, Madison, USA) were performed. PCR amplification was conducted using the Eastep qPCR Master Mix (LS2062, Promega, Madison, USA). Relative gene expression was calculated using the Δ2^−ΔΔCt^ method, with β-actin as the reference gene.

### Pyruvate and lactate measurement

The contents of the two substances in the cell supernatant were detected using a pyruvate kit (E-BC-K130-M, Elabscience, Wuhan, China) and a lactate kit (E-BC-K044-M, Elabscience, Wuhan, China). The detection solution was prepared according to the kit instructions, and the Optical density (OD) values were measured at 505 nm and 530 nm using a microplate reader. The corresponding concentrations of pyruvate and lactate were calculated based on the standard curve.

### CCK8 reagent kit

Prepare a cell suspension and inoculate it into a 96-well plate, with an initial cell concentration of 5000 cells/well. After the cells adhere, add different concentrations of drugs to each well and incubate in a 37 °C incubator for the corresponding time. Then, add 10 µl of CCK-8 enhancement solution (MA0218, meilunbio, Dalian Meilun Biotechnology Co., Ltd) to each well. Incubate in the incubator for 1–3 h and measure the absorbance at 450 nm.

### Scanning electron microscopy (SEM)

Collect the cell pellet, resuspend it, and seed the cells on sterile coverslips in a culture dish, then add electron microscopy fixative to the dish. After fixation, dehydration, and drying, attach the specimen to a metal stub using carbon tape, and then coat with gold for 30 s. Observe and photograph using a scanning electron microscope. Collect images and analyze them.

### Dual immunofluorescence staining

Permeabilize the cell slides, block, and incubate with a mixed reagent containing two primary antibodies (ESRRA, 1:50, PA5-35348, ThermoFisher, MA, USA; LDHA, 2 μg/ml, 10E6AA9, Abcam, London, UK), then add the corresponding fluorescent secondary antibodies. Drop in the DAPI solution and incubate, then add the anti-fade reagent and cover with a coverslip. Perform microscopic detection and image acquisition using a laser confocal microscope at a magnification of 800×.

### In vivo mouse xenograft assay

Female BALB/c (RRID: MGI:2683685) nude mice (*N* = 30, 4–5 weeks old) were purchased from Zhejiang Weitong Lihua Experimental Animal Technology Co., Ltd. The ES-2 cell line was resuspended in Matrigel and transplanted into the axilla of female BALB/c mice. XCT790 (B3238, APExBIO, USA) is a reverse agonist of estrogen-related receptor alpha (ERRα). One week after tumor cell transplantation, the mice were randomly divided into six groups: ES2 group (injected with PBS into ES2 xenograft mice every three days as a negative control), ES2 + XCT790 treatment group (injected intraperitoneally with 5 mg/kg XCT790 into xenograft mice every three days for 28 days), ES2 + Carb treatment group (injected intraperitoneally with 10 mg/kg Carb into xenograft mice once a week for 28 days), ES2 + PTX treatment group (injected intraperitoneally with 10 mg/kg PTX into xenograft mice every three days for 28 days), ES2 + Carb + XCT790 treatment group (injected intraperitoneally with 10 mg/kg Carb once a week and 5 mg/kg XCT790 every three days into xenograft mice for 28 days), ES2 + PTX + XCT790 treatment group (injected intraperitoneally with 10 mg/kg PTX and 5 mg/kg XCT790 every three days for 28 days). Tumor size was measured every 4 days, and tumor volume was calculated using the following formula: tumor volume = (length × width × width)/2. At the end of the study, the mice were anesthetized and euthanized. Tumor tissue and other organs, including the liver, spleen, kidneys, and heart, were carefully excised for further study. This study was approved by the Institutional Animal Care and Use Committee (IACUC) of Fujian Medical University (Ethics Approval No. FJMU 2025-0028).

### Immunohistochemical staining

Immunohistochemical staining was performed according to standard procedures. Rabbit polyclonal antibodies against ERR α (1:100; ab137489, Abcam, London, UK), LDHA (1:1000, A1146, ABclonal, Wuhan, China), and GSDMD (1:500, E9S1X, Cell Signaling Technology, Massachusetts, USA) were used. Three low-power fields (×100) were randomly selected under a microscope, and the positive staining intensity was scored as strong, medium, weak, and negative, corresponding to scores of 3, 2, 1, and 0, respectively. The proportion of stained cells was categorized as 76% ~ 100%, 51% ~ 75%, 26% ~ 50%, and 0 ~ 25%, with corresponding scores of 4, 3, 2, and 1. The product of the intensity and percentage of positively stained cells is the immunohistochemical score of the sample. Samples were classified into four groups based on their immunoreactivity scores: 0, negative (−); 1–4, weak positive (+); 5–8, positive (++); and 9–12, strong positive (+++).

### ATP/ADP ratio

The ATP/ADP ratio of cells was measured with ATP/ADP Ratio Chemiluminescence Assay Kit (E-BC-F004, Elabscience, Wuhan, China) according to the manufacturer’s instructions. This approach depends on luciferase catalyzing the reaction of ATP with luciferin to produce a detectable luminescent signal. In brief, samples containing 1 × 10^6^ cells were lysed via exposure to 150 μL nucleotide-releasing buffer over a 10-minute incubation period in a boiling water bath. Subsequently, 100 μL ATP monitoring enzyme solution was introduced into each well of a 96-well plate to establish the baseline luminescence reading labeled L1. Immediately thereafter, 20 μL of the resulting cell lysate was added and the luminescence intensity L2 was promptly recorded, yielding the ATP level as the difference L2 minus L1. Following a further 20-min interval, luminescence L3 was acquired. Next, 50 μL ADP convertase reagent was dispensed into the wells; after 6 min of equilibration at ambient temperature, the luminescence reading L4 was obtained. ADP content was calculated from L4 minus L3, and the final ATP/ADP ratio was derived by dividing (L2 − L1) by (L4 − L3).

### Statistical analysis

Statistical analysis was performed using R (New England, RRID: SCR_001905), GraphPad Prism 9 and ImageJ (RRID: SCR_003070) software packages were used for plotting. Experiments were repeated at least three times. Data distribution and homogeneity of variance were assessed before statistical analysis, and appropriate statistical tests were selected accordingly. Continuous variables were presented as mean ± SD or median with interquartile range (IQR). Categorical variables were expressed as percentages. Comparisons of categorical variables were performed using the chi-square test or Fisher’s exact test. Student’s *t* test was used for comparisons between two groups, while one-way analysis of variance (ANOVA) was applied for multiple group comparisons, including analyses of western blotting, immunohistochemistry, and immunofluorescence data. All statistical analyses use a significance level of *p* < 0.05.

## Result

### Elevated levels of LDHA and ERRα are associated with poor prognosis in cancer patients and play an important role in OC chemoresistance

**Fig. 1:**
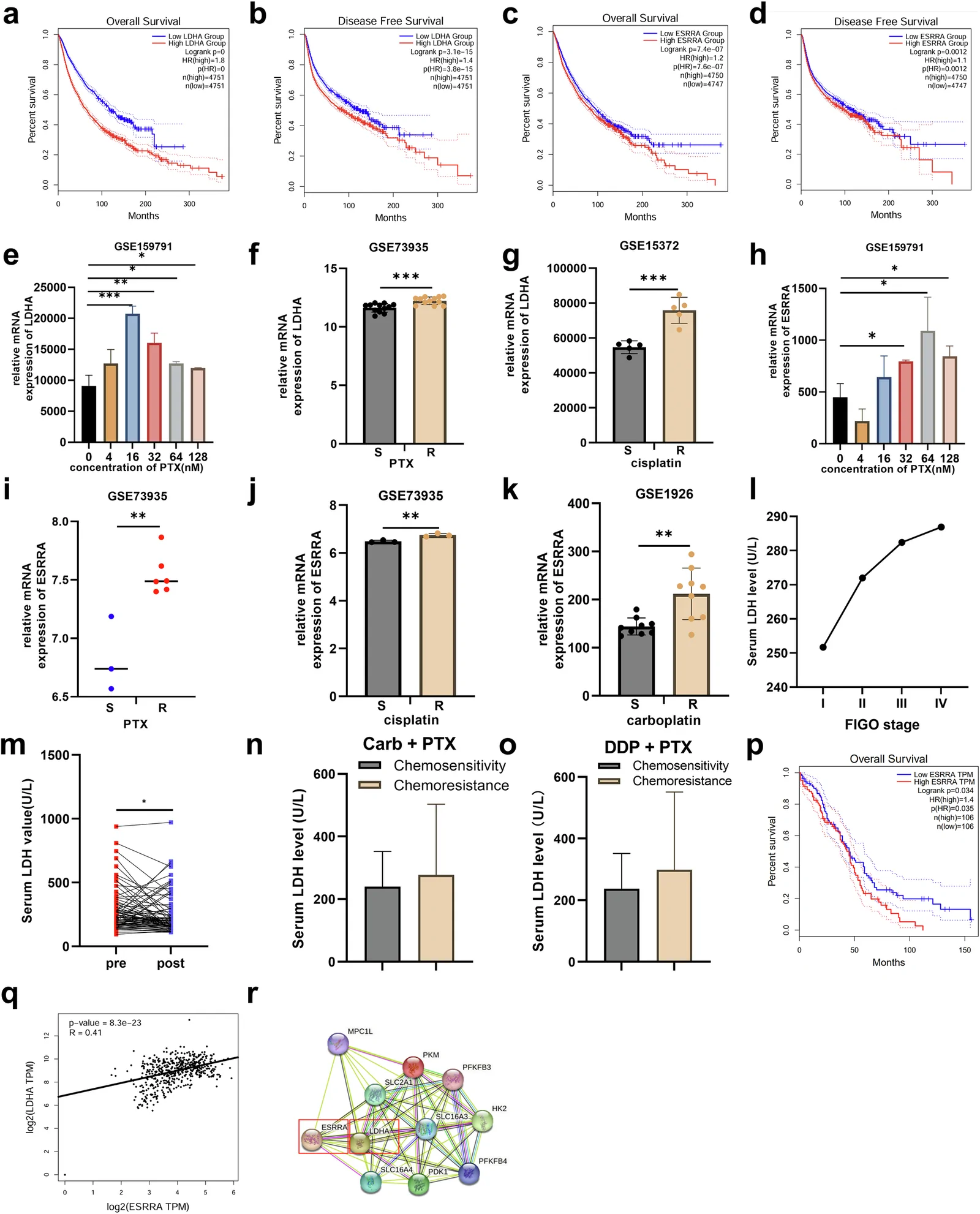
Analysis of the expression of ESRRA and LDHA in tumor cells based on bioinformatics databases. **a**–**d** Impact of high or low expression of LDHA (or ESRRA) on OS or DFS of cancer patients in the GEPIA database. **e**–**k** Expression of LDHA (or ESRRA) in different datasets from the GEO database. **e** In the GSE159791 dataset, A2780 cells were induced with increasing concentrations of PTX (4 nM, 16 nM, 32 nM, 64 nM, 128 nM) to generate PTX-resistant A2780 sublines, analyzing the relative expression of LDHA mRNA. **f** In the GSE73935 dataset, primary human OC cells were induced to develop PTX resistance, analyzing LDHA mRNA expression differences between resistant and sensitive cells (S: sensitive, R: resistant). **g** In the GSE15372 dataset, A2780 cells were induced with increasing DDP concentrations to analyze LDHA mRNA expression differences. **h** In the GSE159791 dataset, relative expression of ESRRA mRNA in different PTX-resistant A2780 sublines. **i** In the GSE73935 dataset, ESRRA mRNA expression differences in PTX-resistant A2780 cells. **j** In the GSE73935 dataset, ESRRA expression differences in DDP-resistant human OC cells. **k** In the GSE1926 dataset, ESRRA mRNA expression differences between Carb-resistant and -sensitive OC tissues. **l**–**o** Clinical data from 124 OC patients at Fujian Provincial Maternity and Child Health Hospital (2005–2023). **l** Serum LDH levels at different FIGO stages. **m** Changes in serum LDH levels before and after surgery (3–5 days pre-surgery, 1st day post-surgery). **n** Serum LDH levels in Carb + PTX-sensitive vs. -resistant patients. **o** Serum LDH levels in DDP + PTX-sensitive vs. -resistant patients. **p** Impact of high or low ESRRA expression on OS in OC patients in the GEPIA database. **q** Correlation between ESRRA and LDHA in OC and normal ovarian tissues in the GEPIA database. **r** Protein interaction network related to ESRRA and LDHA in the STRING database. *p < 0.05; **p < 0.01; ***p < 0.001. GEPIA Gene Expression Profiling Interactive Analysis, TCGA The Cancer Genome Atlas, GTEx Genotype-Tissue Expression, GEO Gene Expression Omnibus, OS Overall Survival, DFS Disease-free Survival, FIGO International Federation of Gynecology and Obstetrics, STRING Search Tool for the Retrieval of Interacting Genes/Proteins, DDP cisplatin, Carb carboplatin, PTX paclitaxel.

Previous studies have reported that both ERRα and LDHA are associated with tumorigenesis and poor prognosis in multiple cancers [19–22]. We performed bioinformatics analyses using several public databases to explore their clinical relevance. GEPIA, which integrates RNA-seq data from TCGA and GTEx (9736 tumors and 8587 normal samples)[23], revealed that elevated ESRRA and LDHA mRNA expression levels correlated with shorter OS and DFS across cancers (Fig. 1a–d). Analysis of GEO datasets further demonstrated that LDHA expression increased in PTX-resistant A2780 sublines (GSE159791), primary OC cells (GSE73935), and DDP-resistant A2780 cells (GSE15372) (Fig. 1e–g). Similarly, ESRRA mRNA levels were significantly upregulated in PTX-resistant (GSE159791, GSE73935) and DDP-resistant OC cells (GSE73935) (Fig. 1h–j) and in tumor tissues from Carb-resistant patients (GSE1926) (Fig. 1k). Clinical data from 124 OC patients at Fujian Provincial Maternity and Child Health Hospital (2005–2023) showed a trend of increasing serum LDH levels with a higher FIGO stage (Fig. 1l). Postoperative LDH levels were significantly lower than preoperative levels (Fig. 1m), suggesting that LDH is a potential tumor marker for OC. In addition, drug sensitivity testing of 105 OC tissues revealed higher serum LDH levels in chemoresistant versus chemosensitive patients under the DDP + PTX or Carb + PTX regimens (Fig. 1n, o, Table S1-S2), indicating a correlation between elevated LDH and platinum-based chemoresistance. Consistent with these findings, GEPIA analysis showed that high ESRRA expression was associated with poorer OS in OC patients (Fig. 1p). ESRRA expression was positively correlated with LDHA in both OC and normal ovarian tissues (Fig. 1q), and STRING analysis suggested a potential protein–protein interaction between them (Fig. 1r). Collectively, ERRα and LDHA are overexpressed in chemoresistant OC cells and tissues, and their interactions may contribute to OC progression and poor prognosis.

### ERRα is an upstream transcription factor of LDHA that positively regulates its expression

**Fig. 2:**
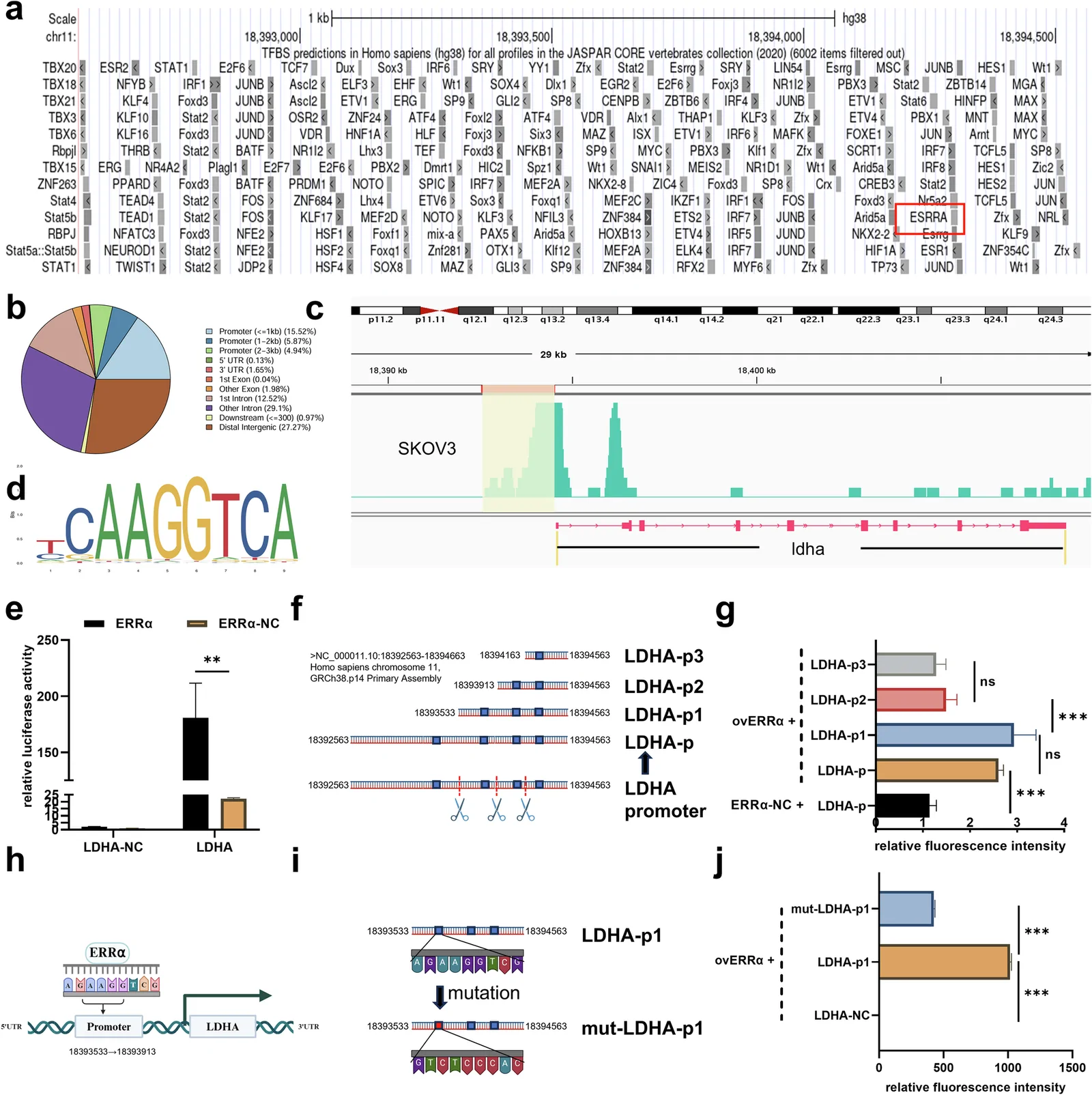
ERRα transcriptional regulation of LDHA. **a** Predicted upstream transcription factors of LDHA in the UCSC database. **b** Distribution map of ERRα peaks in gene functional regions analyzed by CUT&Tag and sequencing. **c** Evaluation of LDHA promoter occupancy in SKOV3 cells. ERRα was immunoprecipitated using anti-Flag antibody. **d** Predicted gene promoter sequences that transcription factor ERRα may bind to from the JASPAR database; the larger the base letter at each site, the greater the likelihood of that base appearing. **e**–**j** Dual-luciferase reporter gene assay, using the ratio of firefly luciferase to Renilla luciferase as the final fluorescence intensity. **e** 293T cells co-transfected with the ERRα transcription factor, firefly luciferase reporter gene-marked LDHA promoter, and Renilla luciferase control. The negative control (NC) group is LDHA-NC + ERRα-NC, LDHA + ERRα-NC, and LDHA-NC + ERRα. **f** The promoter sequence of LDHA (LDHA-p) was cut into truncated forms containing different potential binding sites: LDHA-p1, LDHA-p2, and LDHA-p3. **g** 293T cells were transfected with the ERRα transcription factor, different LDHA truncations, and Renilla luciferase; the negative control group was LDHA-p + ERRα-NC. **h** ERRα binds to the 5′-AGAAGGTCG-3′ region on the LDHA promoter, promoting LDHA transcription. **i** The binding site on LDHA-p1 was mutated, named mut-LDHA-p1. **j** 293T cells were transfected with mut-LDHA-p1, LDHA-p1, and LDHA-NC separately. *n* = three independent experiments. *p < 0.05; **p < 0.01; ***p < 0.001. UCSC University Of California Santa Cruz, CUT&Tag Cleavage Under Targets and Tagmentation.

ERRα, an orphan nuclear receptor involved in cellular energy metabolism [24], plays a key role in glucose metabolism [18, 25]. Using the UCSC Genome Browser, ESRRA was identified as an upstream transcription factor of LDHA (Fig. 2a). CUT&Tag analysis revealed ERRα enrichment within the gene functional regions (Fig. 2b) and direct binding to the LDHA promoter (Fig. 2c). The JASPAR database [26] predicted an ERRα-binding motif (“TCAAGGTCA”) on the LDHA promoter (Fig. 2d), and four potential binding sites were identified. Dual-luciferase reporter assays confirmed that ERRα binding enhanced LDHA transcription (Fig. 2e). To locate the specific binding site, truncated LDHA promoter constructs (LDHA-p1, LDHA-p2, and LDHA-p3) were generated using restriction enzymes (Fig. 2f). Transfection into 293T cells showed significantly decreased luciferase activity in LDHA-p2 compared with LDHA-p1, whereas LDHA-p2 and LDHA-p3 exhibited similar activity (Fig. 2g), indicating a key cis-regulatory element within chr11:18393533–18393913. Based on JASPAR predictions, the ERRα binding sequence “AGAAGGTCG” was identified as the active site (Fig. 2h). Mutation of this site (mut-LDHA-p1) markedly reduced reporter activity compared to that of LDHA-p1 (Fig. 2j), confirming that ERRα directly binds to this promoter region to activate LDHA transcription.

### ERRα/LDHA regulates glycolytic metabolic reprogramming

**Fig. 3:**
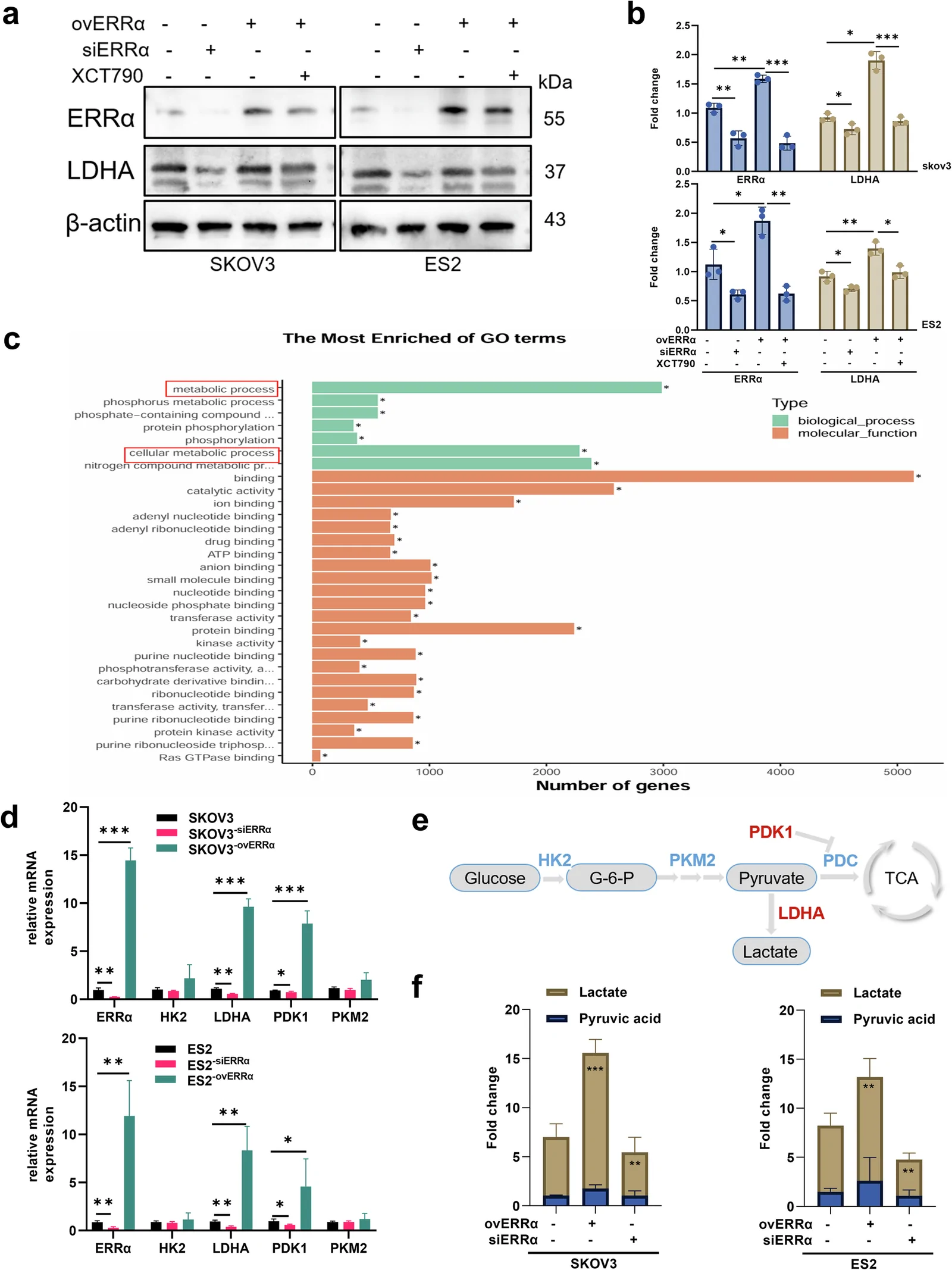
ERRα enhances glycolysis in OC cells and promotes lactate production. **a**, **b** Collect SKOV3 (or ES2) cells overexpressing or inhibiting ERRα, as well as ERRα-overexpressing cells treated with XCT790, extract total protein, and analyze using WB. The control group is wild-type SKOV3 (or ES2) cells. β-actin is used as a reference. Data represent the average of three independent experiments. **c** Perform GO analysis on the CUT&Tag sequencing results. **d** Collect precipitates from SKOV3, SKOV3^-ovERRα^, SKOV3^-siERRα^, ES2, ES2^-ovERRα^, and ES2^-siERRα^ cells, extract total RNA, reverse transcribe to cDNA, and then perform q-PCR to compare the expression differences of glycolysis-related genes (HK2, LDHA, PDK1, PKM2), with β-actin as a reference. **e** The process of glucose entering the cell for glycolysis and the tricarboxylic acid cycle, along with the catalytic enzymes involved. **f** Collect the supernatant from SKOV3, SKOV3^-ovERRα^, SKOV3^-siERRα^, ES2, ES2^-ovERRα^, and ES2^-siERRα^ cells, and measure the levels of pyruvate and lactate. *n* = three independent experiments. *p < 0.05; **p < 0.01; ***p < 0.001. WB Western Blot, CUT&Tag Cleavage Under Targets and Tagmentation, GO Gene Ontology.

To explore the interaction between ERRα and LDHA in OC cells, SKOV3 and ES2 cells were transfected with lentiviruses to generate stable overexpression and knockdown lines after puromycin selection: SKOV3^-ovERRα^, SKOV3^-siERRα^, ES2^-ovERRα^, and ES2^-siERRα^. WB confirmed effective ERRα modulation with corresponding changes in LDHA expression (Fig. 3a, b). Treatment with the ERRα inverse agonist XCT790 reduced ERRα expression in SKOV3^-ovERRα^ and ES2^-ovERRα^ cells. Notably, LDHA protein expression followed the same trend as ERRα expression. GO analysis of CUT&Tag indicated that ERRα-regulated genes were enriched in energy metabolism pathways (Fig. 3c). qPCR analysis revealed that ERRα overexpression upregulated multiple glycolysis-related genes, including HK2, LDHA, PDK1, and PKM2, whereas ERRα knockdown produced the opposite effect. Among these ERRα-regulated glycolytic genes, LDHA and PDK1 exhibited pronounced expression changes, with LDHA showing a greater magnitude of alteration than PDK1 (Fig. 3d). Mechanistically, LDHA directly catalyzes the conversion of pyruvate to lactate, whereas PDK1 indirectly influences lactate metabolism by inhibiting PDC activity and restricting pyruvate entry into the TCA cycle (Fig. 3e). Consistent with these findings, ERRα overexpression increased pyruvate and lactate production, while ERRα knockdown reduced both metabolites, with a more significant change observed in lactate levels (Fig. 3f). Together, these results suggest that ERRα promotes glycolytic reprogramming in ovarian cancer cells, primarily by enhancing LDHA-associated lactate production.

### ERRα enhances the drug resistance of OC cells

**Fig. 4:**
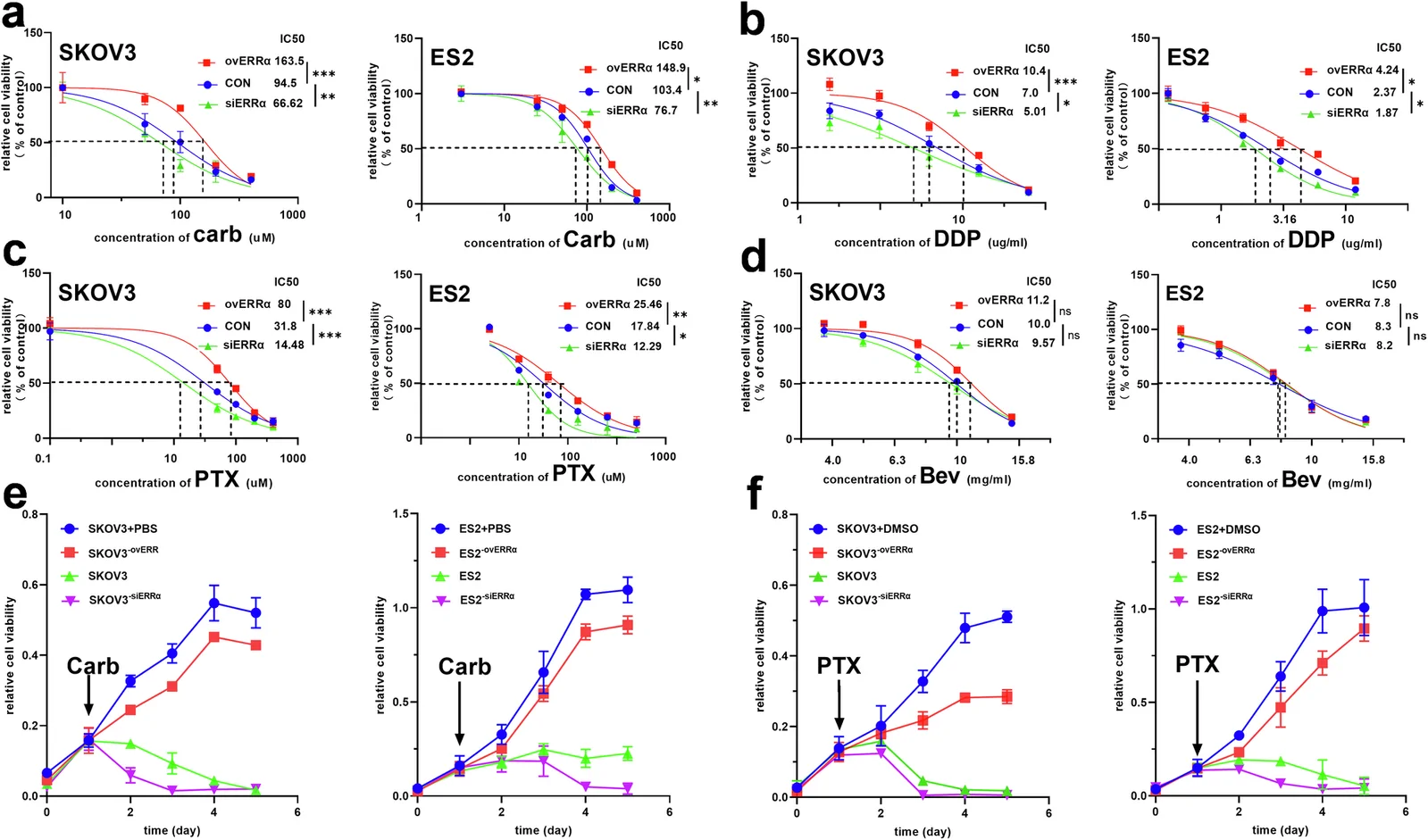
ERRα promotes the resistance of OC cells to the cytotoxic effects of platinum-based drugs and paclitaxel. **a**–**f** Cell viability was assessed using the CCK8 method after different treatments. **a**–**d** SKOV3 and ES2 cells were treated with different concentrations of Carb, DDP, PTX, and Bev for 48 h, and cell viability was measured to calculate IC50. **e**, **f** Growth curves of SKOV3 and ES2 cells. **e** After 24 h of cell plating, 95 µM or 103 µM carboplatin was added as treatment, with SKOV3 or ES2 cells treated with PBS as control. **f** After 24 h of cell plating, 32 µM or 18 µM PTX was added as treatment, with SKOV3 or ES2 cells treated with DMSO as control. *n* = three independent experiments. *p < 0.05; **p < 0.01; ***p < 0.001. Carb carboplatin, PTX paclitaxel, Bev bevacizumab, DDP cisplatin.

ERRα has been reported to contribute to DDP resistance in EC [18]. According to the National Comprehensive Cancer Network (NCCN), Carb combined with PTX is the first-line chemotherapy for epithelial OC, and bevacizumab (Bev) is recommended as class I maintenance therapy [27]. To explore whether ERRα mediates drug resistance in OC cells, we treated SKOV3, SKOV3^-ovERRα^, SKOV3^-siERRα^, ES2, ES2^-ovERRα^, and ES2^-siERRα^ cells with increasing concentrations of Carb for 48 h, followed by CCK8 assays to generate dose–response curves and calculate IC50 values (Fig. 4a). The IC50 values for Carb were 94.5, 163.5, and 66.62 µM for SKOV3, SKOV3^-ovERRα^, and SKOV3^-siERRα^, respectively, and 103.4, 148.9, and 76.7 µM for ES2, ES2^-ovERRα^, and ES2^-siERRα^, respectively. We also tested the effects of DDP, PTX, and Bev (Fig. 4b–d). For DDP, the IC50 values were 7, 10.4, and 5.01 µg/ml in SKOV3, SKOV3^-ovERRα^, and SKOV3^-siERRα^, and 2.37, 4.24, and 1.87 µg/ml in ES2, ES2^-ovERRα^, and ES2^-siERRα^, respectively (Fig. 4b). For PTX, IC50 values were 31.8, 80, and 14.48 µM in SKOV3, SKOV3^-ovERRα^, and SKOV3^-siERRα^, and 17.84, 25.46, and 12.29 µM in ES2, ES2^-ovERRα^, and ES2^-siERRα^, respectively (Fig. 4c). For Bev, IC50 values showed no significant differences among the groups (10, 11.2, and 9.57 mg/ml in SKOV3, SKOV3^-ovERRα^, and SKOV3^-siERRα^; 8.3, 7.8, and 8.2 mg/ml in ES2, ES2^-ovERRα^, and ES2^-siERRα^) (Fig. 4d). These results suggest that ERRα overexpression increased the IC50 of Carb and PTX in SKOV3 and ES2 OC cells, while silencing ERRα decreased the IC50 of these drugs. However, ERRα regulation had no significant effect on the IC50 of Bev. Time-course assays further confirmed these trends: at the IC50 dose of Carb (94.5 µM), ERRα overexpression mitigated drug-induced growth inhibition in both SKOV3 and ES2 cells, whereas ERRα knockdown enhanced cytotoxicity (Fig. 4e). Likewise, ERRα overexpression improved PTX tolerance, while ERRα silencing sensitized cells to PTX-induced cell death (Fig. 4f). In summary, ERRα confers resistance against the cytotoxic effects of Carb and PTX in OC cells.

### ERRα influences pyroptosis in OC cells

**Fig. 5:**
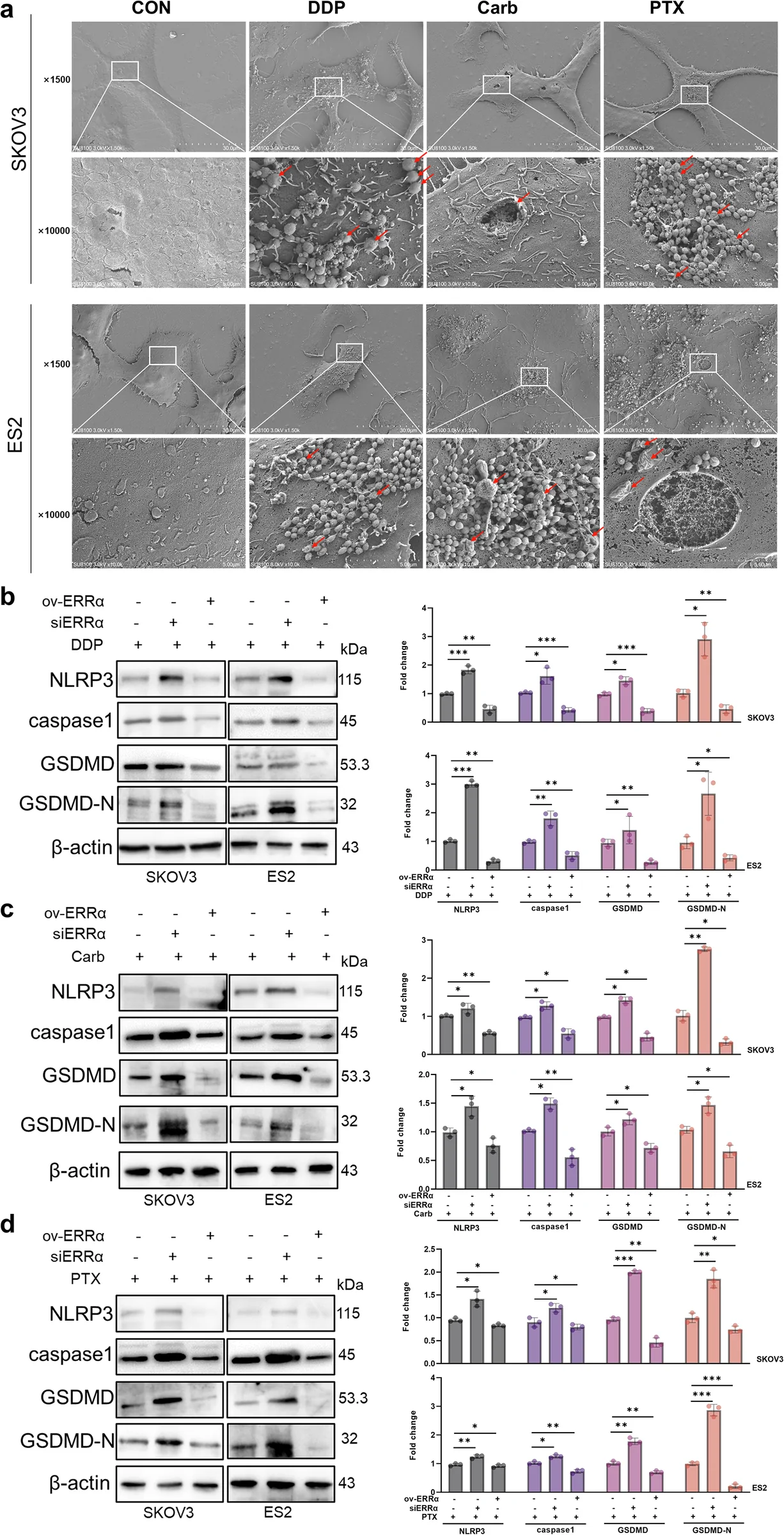
ERRα promotes OC cell resistance to platinum and paclitaxel-induced pyroptosis. **a** Cells were collected, prepared, and observed under a scanning electron microscope for membrane swelling and perforation. Red arrows mark pyroptotic bubbles on the cell membrane. The normal culture medium group was used as a control. SKOV3 cells were treated with 7 μg/ml DDP, 95 μM Carb, and 32 μM PTX for 24 h before preparation for observation. ES2 cells were treated with 2 μg/ml DDP, 103 μM Carb, and 18 μM PTX for 24 h before preparation for observation. **b**–**d** Cells were collected, proteins extracted, and WB experiments conducted to compare the expression differences of NLRP3, caspase-1, GSDMD, and GSDMD-N in cells with different ERRα expression levels. β-actin was used as a reference. Data represent three independent experiments and are expressed as mean values. **b** SKOV3 and ES2 cells were treated with 7 μg/ml and 2 μg/ml DDP for 24 h, respectively. **c** SKOV3 and ES2 cells were treated with 95 μM and 103 μM Carb for 24 h, respectively. **d** SKOV3 and ES2 cells were treated with 32 μM and 18 μM PTX for 24 h, respectively. *n* = three independent experiments. *p < 0.05; **p < 0.01; ***p < 0.001. WB Western Blot, Carb carboplatin, PTX paclitaxel, DDP cisplatin.

Platinum compounds induce DNA damage–mediated cell death, whereas PTX inhibits mitosis; both can trigger pyroptosis in tumor cells [28]. To assess this phenomenon in OC, SKOV3 and ES2 cells were treated with DDP, Carb, or PTX at their IC50 concentrations for 24 h and examined using SEM. Compared with the controls, drug-treated cells exhibited characteristic pyroptotic morphology, including cell swelling, membrane rupture, and pore formation (Fig. 5a) [29]. Given that NLRP3/caspase-1/GSDMD signaling plays a central role in pyroptosis [30], we evaluated these proteins in SKOV3, SKOV3^-ovERRα^, SKOV3^-siERRα^, ES2, ES2^-ovERRα^, and ES2^-siERRα^ cells treated with DDP, Carb, or PTX for 24 h at their corresponding IC50 concentrations (Fig. 5b–d). In both SKOV3 and ES2 cells, ERRα overexpression decreased the expression of NLRP3, caspase-1, GSDMD, and GSDMD-N in response to DDP, Carb, or PTX. Conversely, ERRα knockdown increases the expression of these molecules.

**Fig. 6:**
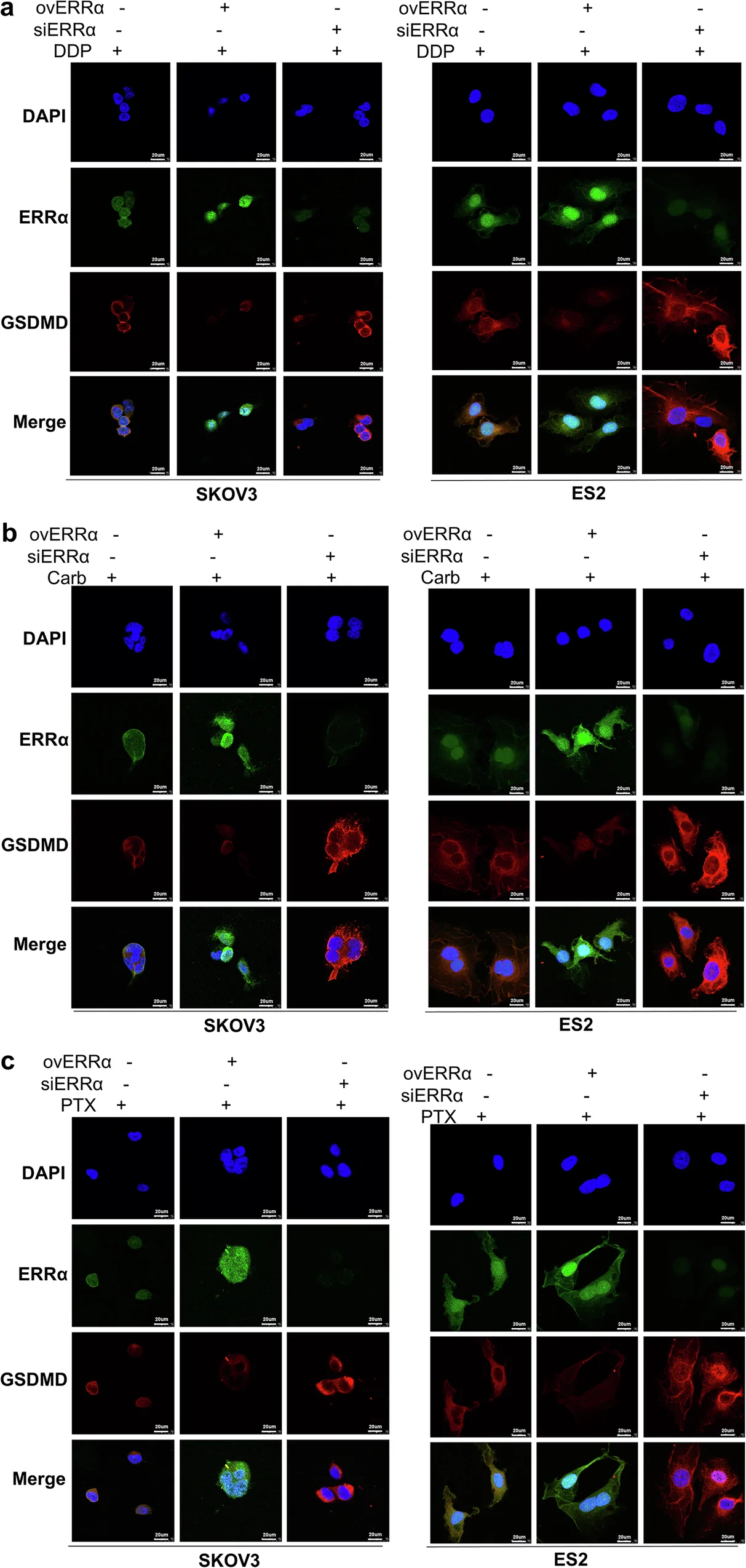
Immunofluorescence shows that ERRα promotes OC cell resistance to platinum‑ and paclitaxel‑induced pyroptosis. **a**–**c** Representative images of cell immunofluorescence staining expressing ERRα and GSDMD were taken using a laser confocal microscope, with blue DAPI fluorescent dye marking the cell nuclei, green fluorescence marking ERRα, and red fluorescence marking GSDMD, using wild-type cells as a control. **a** SKOV3 and ES2 cells were treated with 7 μg/ml and 2 μg/ml DDP for 24 h, respectively. **b** SKOV3 and ES2 cells were treated with 95 μM and 103 μM Carb for 24 h, respectively. **c** SKOV3 and ES2 cells were treated with 32 μM and 18 μM PTX for 24 h, respectively. *n* = three independent experiments. Carb carboplatin, PTX paclitaxel, DDP cisplatin.

The immunofluorescence assays further corroborated these findings (Fig. 6a–c). Overexpression of ERRα (indicated by increased green fluorescence) corresponded to a significant reduction in the red fluorescence intensity (reflecting reduced GSDMD expression). Conversely, ERRα inhibition (indicated by decreased green fluorescence) resulted in a marked increase in the red fluorescence intensity (indicating enhanced GSDMD expression). In conclusion, ERRα attenuates the production of pyroptosis-related molecules and inhibits pyroptosis induced by Carb, DDP, and PTX in OC cells.

### ERRα regulates LDHA to influence cell pyroptosis resistance

**Fig. 7:**
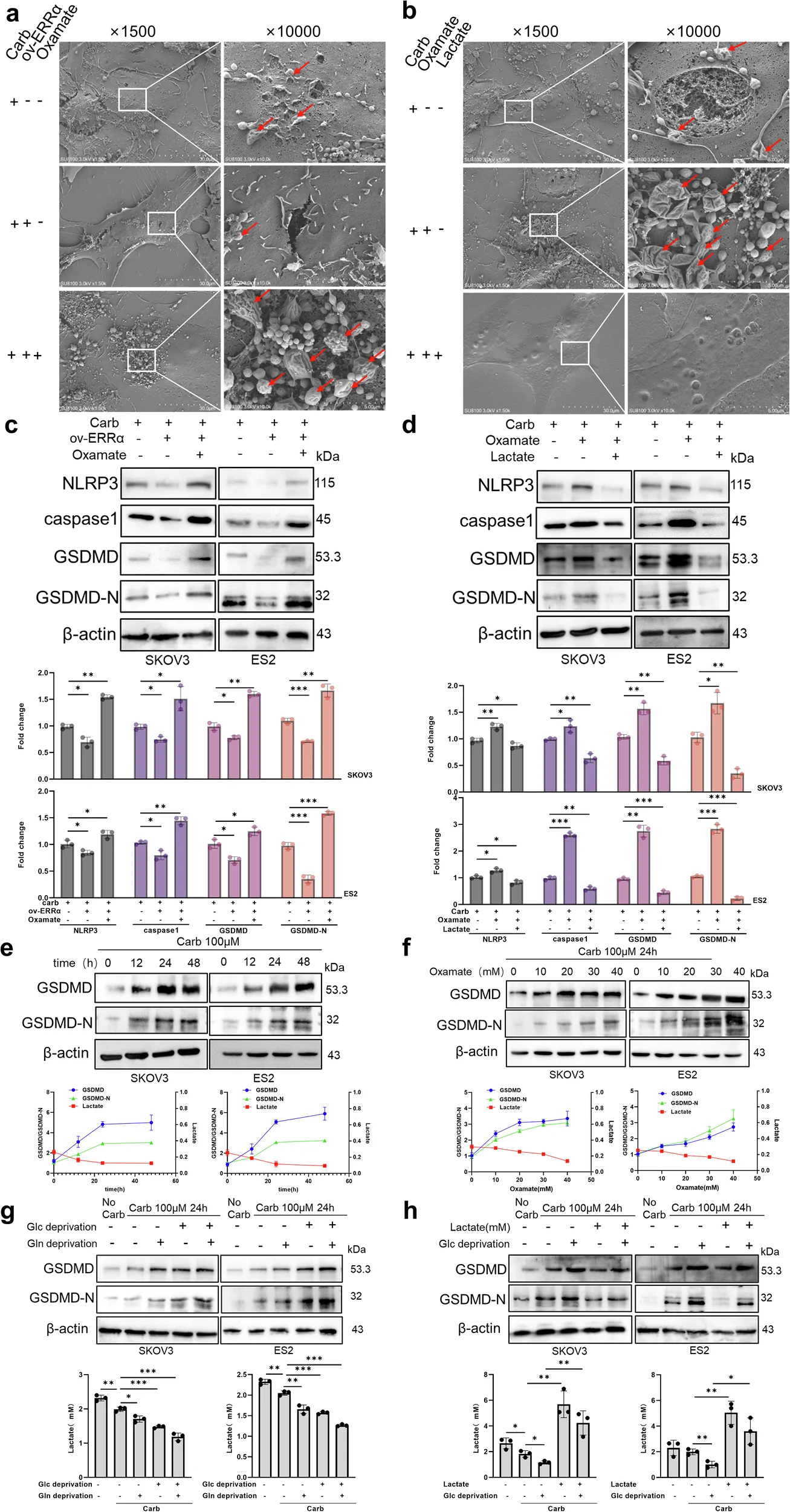
ERRα regulates glycolytic product lactate resistance to cell pyroptosis. **a**, **b** Cells were analyzed for membrane swelling and perforation using SEM. Red arrows mark pyroptotic bubbles on the cell membrane. **a** ES2 and ES2^-ovERRα^ cells were treated with 103 µM Carb for 24 h, with an additional group of ES2^-ovERRα^ cells treated with 103 µM Carb and 30 mM Oxamate (LDHA inhibitor) for 24 h. **b** All ES2 cells were treated with 103 µM Carb for 24 h: control, 30 mM Oxamate, or 30 mM Oxamate plus 50 mM Lactate. **c**–**h** Cell pellets were collected, proteins extracted, and WB experiments performed with β-actin as a reference. **c** ES2 and ES2^-ovERRα^ cells were treated with 103 µM Carb for 24 h, with an additional group treated with 103 µM Carb and 30 mM Oxamate. SKOV3 cells were treated similarly. WB detected NLRP3, caspase-1, GSDMD, and GSDMD-N expression. **d** ES2 cells treated with 103 µM Carb for 24 h were grouped as control, 30 mM Oxamate, or 30 mM Oxamate plus 50 mM Lactate, with SKOV3 treated similarly. WB detected NLRP3, caspase-1, GSDMD, and GSDMD-N expression. **e** SKOV3 and ES2 cells were treated with 100 µM Carb for 12 h, 24 h, and 48 h. WB detected GSDMD, GSDMD-N, and lactate levels in supernatants were measured. **f** SKOV3 and ES2 cells were treated with 100 µM Carb for 24 h and 10–40 mM Oxamate. WB detected GSDMD, GSDMD-N, and lactate levels were measured. **g** SKOV3 and ES2 cells treated with 100 µM Carb for 24 h in glucose-free, glutamine-free, and both-free media. WB detected GSDMD, GSDMD-N, and lactate levels were measured. **h** SKOV3 and ES2 cells were treated with 100 µM Carb for 24 h in glucose-free medium, complete medium with 50 mM Lactate, and glucose-free medium with 50 mM Lactate. WB detected GSDMD, GSDMD-N, and lactate levels were measured. *n* = three independent experiments. *p < 0.05; **p < 0.01; ***p < 0.001. WB Western Blot, Carb carboplatin.

To elucidate the role of LDHA and lactate in ERRα-mediated regulation of pyroptosis in OC, ES2 and ES2^-ovERRα^ cells were treated with Carb (103 µM) for 24 h. And cells were treated with or without the LDHA inhibitor oxamate (30 mM). SEM revealed markedly fewer membrane perforations in ES2^-ovERRα^ cells than in ES2 cells, whereas oxamate co-treatment restored membrane damage and swelling (Fig. 7a), suggesting a protective role for lactate against pyroptosis. To verify this, ES2 cells were divided into three groups: control, oxamate (20 mM), and oxamate plus lactate (50 mM). Oxamate enhanced Carb-induced pyroptosis, whereas lactate supplementation rescued this phenotype and reduced membrane perforations (Fig. 7b). Western blot analysis confirmed that ERRα overexpression suppressed NLRP3, caspase-1, GSDMD, and GSDMD-N expression following Carb exposure, with oxamate reversing this effect and lactate attenuating the suppression (Fig. 7c, d). Time-course experiments to analyze protein expression at 0, 12, 24, and 48 h showed an inverse correlation between the lactate level and the expression of GSDMD and GSDMD-N following Carb treatment; lactate decreased while GSDMD and GSDMD-N increased over time (Fig. 7e). Similarly, oxamate reduced lactate production and elevated GSDMD and GSDMD-N expression in a dose-dependent manner (Fig. 7f).

To assess the role of lactate in pyroptosis, SKOV3 and ES2 cells were cultured in glucose- and/or glutamine-free media and treated with 100 µM Carb for 24 h. Nutrient deprivation reduced lactate levels and an increase in the level of cell pyroptosis, with a stronger effect under glucose deprivation, and the lowest levels were observed under combined deprivation (Fig. 7g). Lactate levels were inversely correlated with GSDMD and GSDMD-N expression. Moreover, lactate supplementation in glucose-deprived media reduced the expression of GSDMD and GSDMD-N (Fig. 7h), confirming lactate’s role in modulating pyroptosis.

To determine whether the enhanced pyroptosis observed under glucose deprivation was attributable to cellular energy depletion, we assessed intracellular ATP/ADP ratios under the indicated conditions. The results showed that glucose deprivation alone caused a slight reduction in the ATP/ADP ratio. However, this change did not reach statistical significance compared with the control group (*p* > 0.05), suggesting that cells may maintain energy homeostasis through compensatory glutamine metabolism. In contrast, combined deprivation of glucose and glutamine resulted in a marked decrease in ATP/ADP ratio (*p* < 0.05), consistent with severe bioenergetic stress due to the loss of two major carbon sources (Supplementary Fig. S1a). Notably, despite the absence of a significant reduction in ATP/ADP ratio, glucose deprivation alone significantly increased pyroptosis levels. This observation indicates that pyroptotic activation does not directly correlate with global cellular energy status. To further investigate this, we supplemented exogenous lactate under both normal and glucose-deprived conditions. ATP/ADP ratios remained comparable across all treatment groups, indicating that overall energy homeostasis was largely preserved. However, glucose deprivation markedly enhanced pyroptosis, whereas lactate supplementation effectively attenuated this effect (Supplementary Fig. S1b). So these results suggest that glucose deprivation–induced pyroptosis is not primarily driven by energy depletion, but is more closely associated with reduced lactate production and metabolic reprogramming. Collectively, these findings demonstrated that ERRα inhibits chemotherapy-induced pyroptosis through LDHA-mediated lactate production. Lactate acts as a key effector in ERRα-driven chemoresistance, underscoring the pivotal role of LDHA in regulating pyroptosis during platinum-based treatment of OC.

### In the xenograft animal model, targeted inhibition of ERRα hinders tumor growth and promotes sensitivity to Carb and PTX

**Fig. 8:**
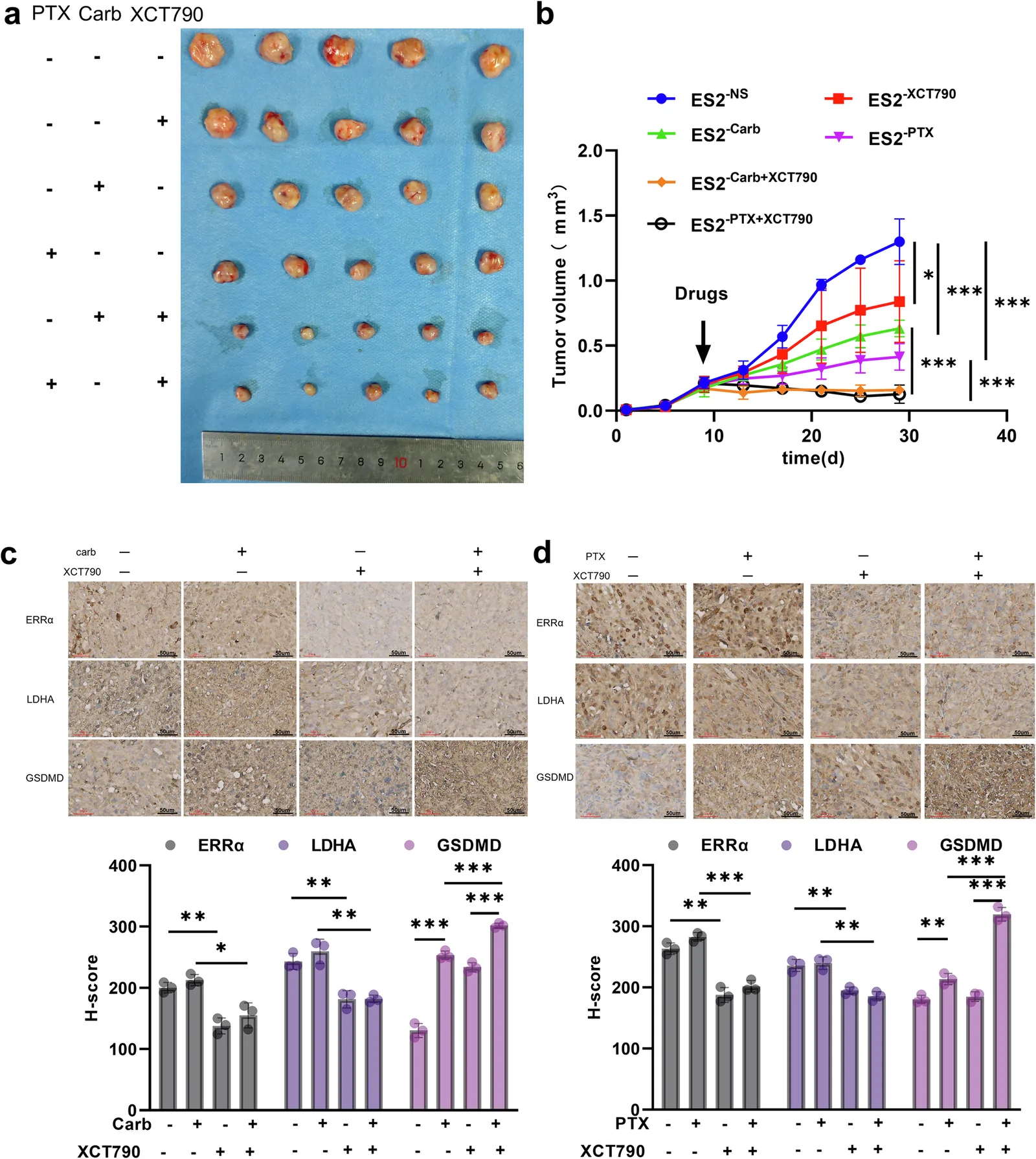
The resistance of ERRα to the cytotoxic effects of Carb and PTX in nude mouse tumor experiments. **a** Representative images of tumors and tumor sizes captured from six treatment groups of BALB/c nude mice at the end of the study. **b** Tumor growth curves for the six treatment groups. **c**, **d** Representative images of immunohistochemistry for ERRα, LDHA, and GSDMD in tumor sections from xenograft mice (each point represents the average of three random fields for each sample). *p < 0.05; **p < 0.01; ***p < 0.001. WB Western Blot, Carb carboplatin, PTX paclitaxel.

To investigate whether in vivo ERRα inhibition promoted pyroptosis via LDHA regulation and enhanced sensitivity to Carb or PTX, we established an ES2 xenograft model in BALB/c nude mice. One week after tumor cell implantation, mice were divided into six groups and treated with intraperitoneal injections of PTX, Carb, or XCT790, depending on the treatment group. Representative tumor images and sizes at the end of the experiment are shown in Fig. 8a. Both Carb and PTX significantly inhibited tumor growth compared with the control, with the combination of Carb/PTX and XCT790 showing the most pronounced effect (Fig. 8b). Tumor growth was slower in the Carb + XCT790 and PTX + XCT790 groups compared with the Carb and PTX alone groups. These results suggest that inhibition of ERRα hinders tumor growth and enhances sensitivity to Carb or PTX. Immunohistochemistry analysis revealed that XCT790 treatment reduced ERRα and LDHA expression (Fig. 8c) while increasing GSDMD levels in the Carb- and PTX-treated groups (Fig. 8d). No significant hepatotoxicity or weight loss was observed in any treatment group. These findings indicate that the inhibition of ERRα promotes pyroptosis by regulating LDHA, affects the GSDMD pathway, and enhances the sensitivity of OC cells to Carb or PTX.

## Discussion

The connection between metabolic reprogramming of tumor cells and drug resistance has been a key research focus in recent years. ERRα, a central regulator of energy metabolism, has been linked to drug resistance in various cancers through different mechanisms [10, 31–35]. Pyroptosis, a form of inflammatory cell death, can be induced in lung cancer cells by both PTX and DDP via the caspase-3/GSDME/GSDME-N pathway, with DDP being more effective at inducing pyroptosis than PTX [28]. Our research also showed that DDP, Carb, and PTX can trigger pyroptosis in OC cells through the NLRP3/caspase-1/GSDMD/GSDMD-N pathway. We observed no significant difference in the level of pyroptosis induced by the platinum drugs versus PTX, which may be due to the use of the corresponding IC50 concentrations of the drugs. Our preliminary studies suggest that ERRα inhibits the sensitivity of EC to platinum drugs by regulating glycolysis and pyroptosis [18]. In addition, we found that ERRα was involved in both platinum and PTX resistance in OC. As Carb + PTX is a first-line chemotherapy regimen for OC, our findings highlight the potential of ERRα in treating drug-resistant OC. Furthermore, we explored the link between glycolysis and pyroptosis, revealing that ERRα-driven lactate production via LDHA inhibits pyroptosis, likely because of the acidic environment induced by lactate. Our study is the first to demonstrate that ERRα promotes lactate production by targeting LDHA, which inhibits DDP- and PTX-induced pyroptosis, contributing to OC cell resistance to these drugs in vivo and in vitro.

Dysregulation of ERRα activity can lead to metabolic disorders and cancer. In 2018, our team identified ERRα as a key regulator of cellular metabolism, particularly in gynecological endocrine-related tumors and energy metabolism [36]. Knockout of ERRα inhibits invasion, metastasis, and angiogenesis in EC, while promoting apoptosis. Both endogenous and exogenous inhibition of ERRα expression and function have significant anticancer effects. In colon cancer cells, ERRα depletion led to a marked reduction in the expression of key genes involved in glycolysis, the tricarboxylic acid cycle, and lipid synthesis. Conversely, upregulation of ERRα increases the expression of enzymes that regulate glycolysis in breast cancer cells [21]. In the OC cell lines SKOV3 and ES2, we found that the overexpression of ERRα significantly enhanced the transcription of LDHA and directly increased the production of lactate, indicating that ERRα promotes the glycolytic process in OC cells. Furthermore, our study, utilizing dual luciferase assays, is the first to show that ERRα directly binds to the promoter of LDHA and positively regulates its expression. This finding contrasts with that of Mirebeau-Prunier et al., who reported that in thyroid cancer cells, ERRα binds to LDHB and negatively regulates its expression [37]. Typically, elevated serum LDH is observed in non-seminomatous tumors; however, we found that serum LDH levels in OC patients significantly decreased postoperatively. Surgical stimulation generally causes a short-term increase in serum LDH, peaking at 34 h for LDH1 and 58 h for LDH2 [38], which rules out surgical effects on serum LDH levels. Elevated serum LDH levels in cancer patients are commonly associated with poor clinical outcomes and drug resistance [20, 39]. Our study also showed that serum LDH levels tended to increase in patients with chemotherapy-resistant OC. These results suggest that LDH levels may have potential value in predicting and monitoring OC recurrence and chemoresistance.

Abnormal glycolytic metabolism is directly linked to chemotherapy resistance in several cancers, including breast and cervical cancer [40–42]. Recently, inducing pyroptosis in tumor cells using drugs has emerged as a potential strategy to overcome chemotherapy resistance, with the added benefits of reducing side effects and activating immune responses, making it a promising approach [43–45]. Pyroptosis is closely associated with the proliferation, invasion, and metastasis of tumor cells, and can significantly affect the efficacy of chemotherapy [46]. Studies have demonstrated that inhibiting ERRα can enhance the efficacy of chemotherapy in tumors [47] and that tumor cell resistance to pyroptosis contributes to chemotherapy resistance [48]. Moreover, promoting pyroptosis has been shown to improve clinical outcomes in cancer patients undergoing anticancer treatment [49]. Our study found that the treatment of SKOV3 and ES2 cells with DDP, Carb, or PTX led to cell membrane swelling and perforation, as observed by SEM. This suggests that pyroptosis may be a common pathway through which chemotherapeutic drugs induce tumor cell death. ERRα, a key regulator of cellular energy metabolism, is involved in critical processes such as tricarboxylic acid cycle [50] and lipid metabolism [51]. Research has shown that glycolytic metabolites, specifically L-lactate and D-lactate, enhance the survival of cervical cancer cells after chemotherapy [52]. Similarly, Barnes et al. demonstrated that lactate promotes resistance to uprosertib in colon cancer cells [53] and that lactate may play a role in tamoxifen resistance in breast cancer cells [54]. These findings align with our results, which suggest that lactate, a glycolytic product, plays a crucial role in tumor cell resistance to pyroptosis and chemotherapeutic drugs.

A limitation of this study is the use of an immunodeficient nude mouse model, which does not fully replicate the tumor immune microenvironment. Although our findings highlight the tumor cell–intrinsic role of the ERRα-LDHA axis in regulating pyroptosis, the potential contribution of immune components requires further investigation using immunocompetent models.

In conclusion, our study demonstrates that ERRα directly targets the 5′-AGAAGGTCG-3′ sequence in the LDHA promoter, thereby promoting glycolytic activity and lactate production. This process inhibits downstream pyroptosis signaling pathways, enhancing OC resistance to both pyroptosis and chemotherapy with Carb or PTX. These findings suggest that ERRα regulation of pyroptosis signaling pathways may represent a novel therapeutic target to overcome chemotherapy resistance in OC, with potential clinical applications to improve treatment outcomes.

## Supplementary information

Table S1 and Table S2

Supplementary Fig. S1

Full and uncropped western blots

## Data Availability

The data generated in this study are available upon request from the corresponding author.
