# Supplementary material for: Metabolic activation of LDHA by ERRα represses inflammasome-dependent pyroptosis and promotes ovarian cancer chemoresistance
Source: Cell Death Dis. 2026 Jun 17;17(1):754. doi: 10.1038/s41419-026-08986-6 (PMC13507210; doi:10.1038/s41419-026-08986-6)
Supplement: Supplementary file 1 [file 41419_2026_8986_MOESM2_ESM.pdf]

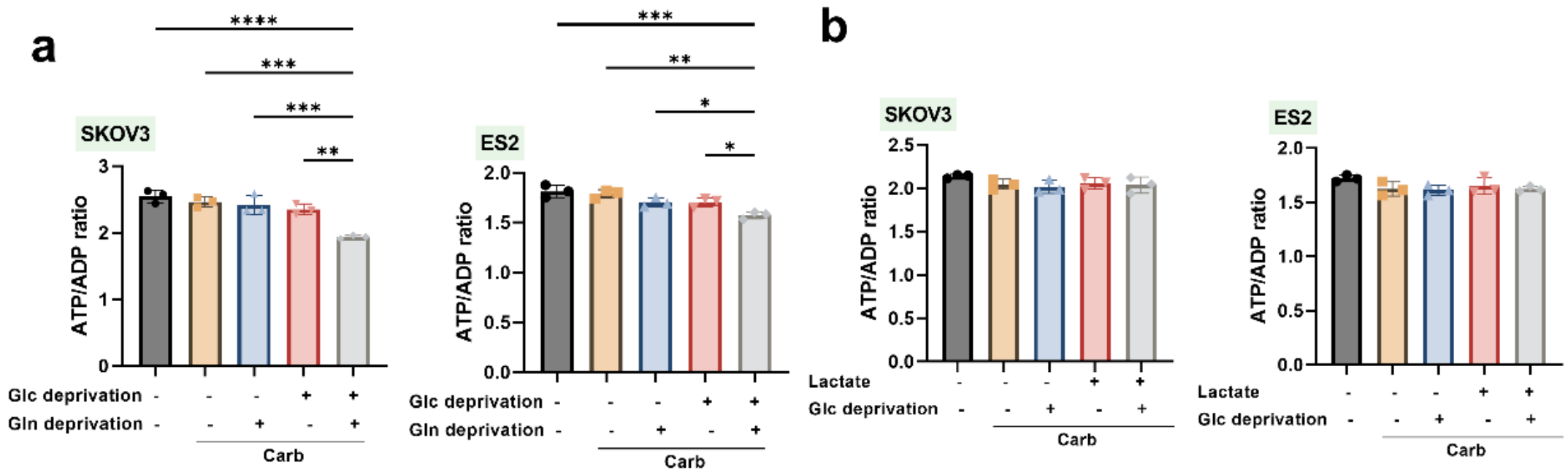

Supplementary Fig. S1 The ATP/ADP ratio of OC cells under different treatment conditions. **a.** SKOV3 and ES2 cells treated with 100 μM Carb for 24 hours in glucose-free, glutamine-free, and both-free media. The ATP/ADP ratio of the cells was measured. **b.** SKOV3 and ES2 cells were treated with 100 μM Carb for 24 hours in glucose-free medium, complete medium with 50 mM Lactate, and glucose-free medium with 50 mM Lactate. The ATP/ADP ratio of the cells was measured. n = three independent experiments. *$p < 0.05$; **$p < 0.01$; ***$p < 0.001$. Abbreviations: Carb, carboplatin; PTX, paclitaxel; DDP, cisplatin.
