# Supplementary material for: Metabolic activation of LDHA by ERRα represses inflammasome-dependent pyroptosis and promotes ovarian cancer chemoresistance
Source: Cell Death Dis. 2026 Jun 17;17(1):754. doi: 10.1038/s41419-026-08986-6 (PMC13507210; doi:10.1038/s41419-026-08986-6)

Fig. 3a

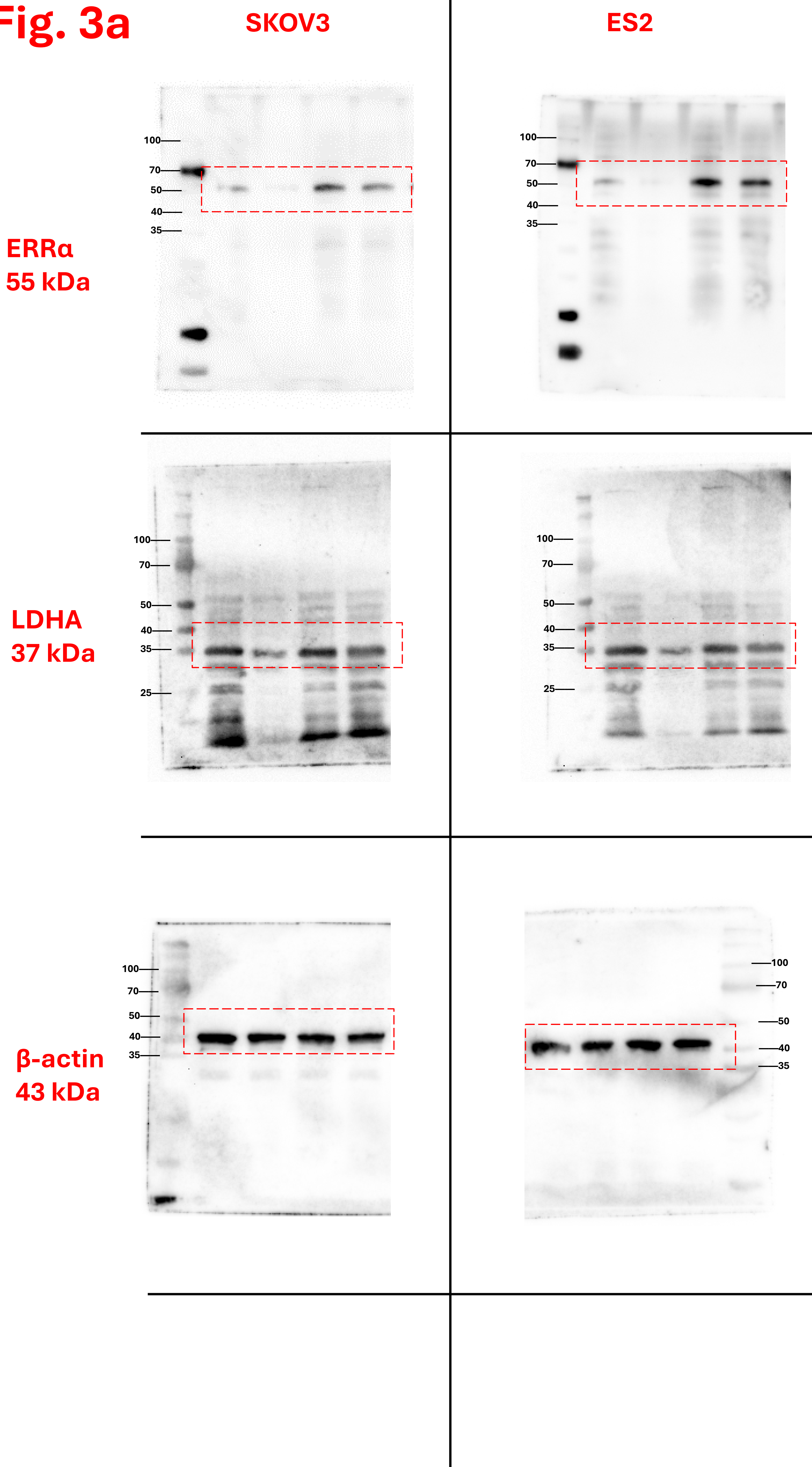

SKOV3
ES2
ERRα
55 kDa
LDHA
37 kDa
β-actin
43 kDa
100
70
50
40
35
25

Fig. 5b

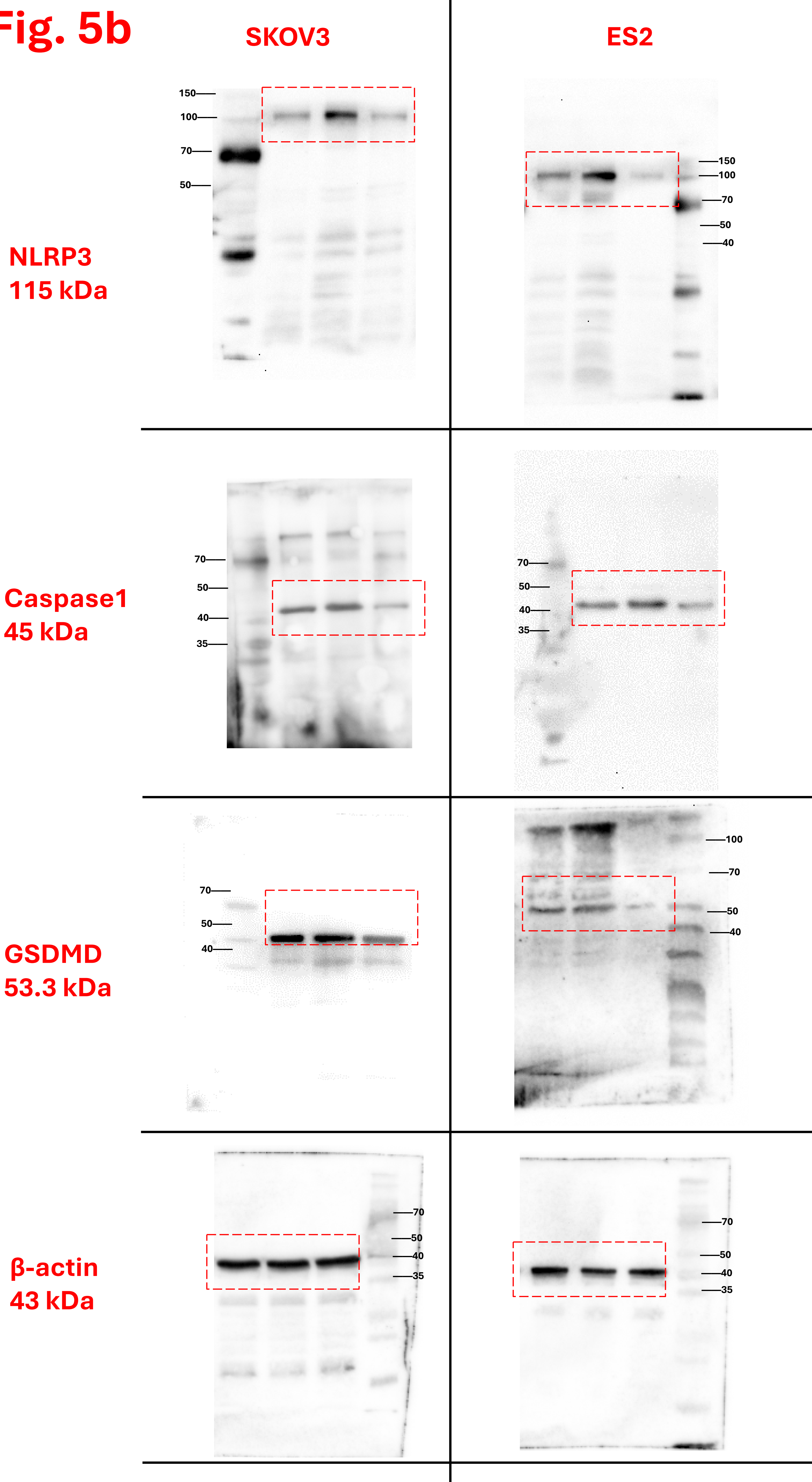

SKOV3
ES2
NLRP3
115 kDa
Caspase1
45 kDa
GSDMD
53.3 kDa
β-actin
43 kDa
150
100
70
50
40
35

Fig. 5c

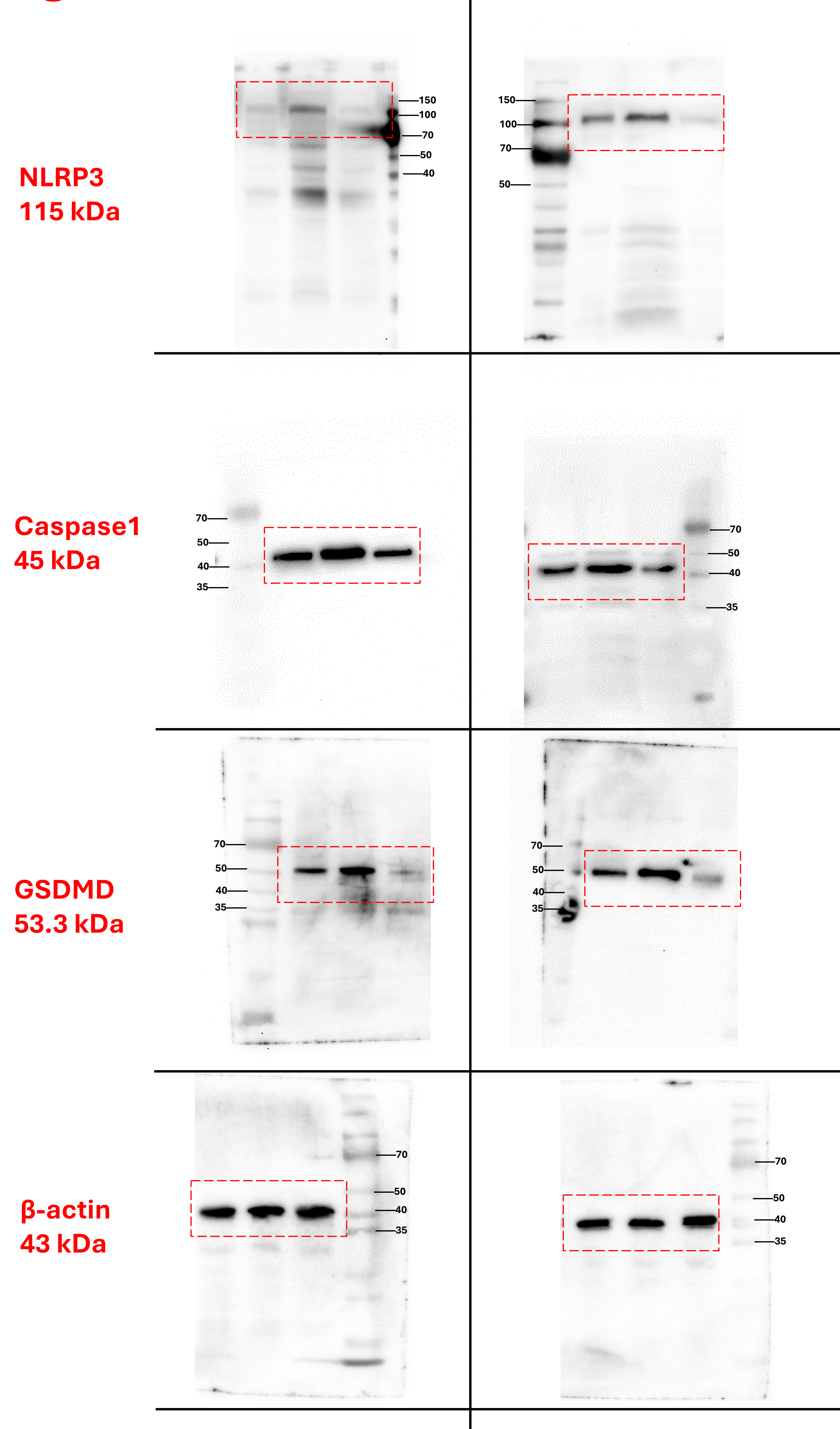

SKOV3
ES2
NLRP3
115 kDa
Caspase1
45 kDa
GSDMD
53.3 kDa
β-actin
43 kDa
150
100
70
50
40
35

Fig. 5d

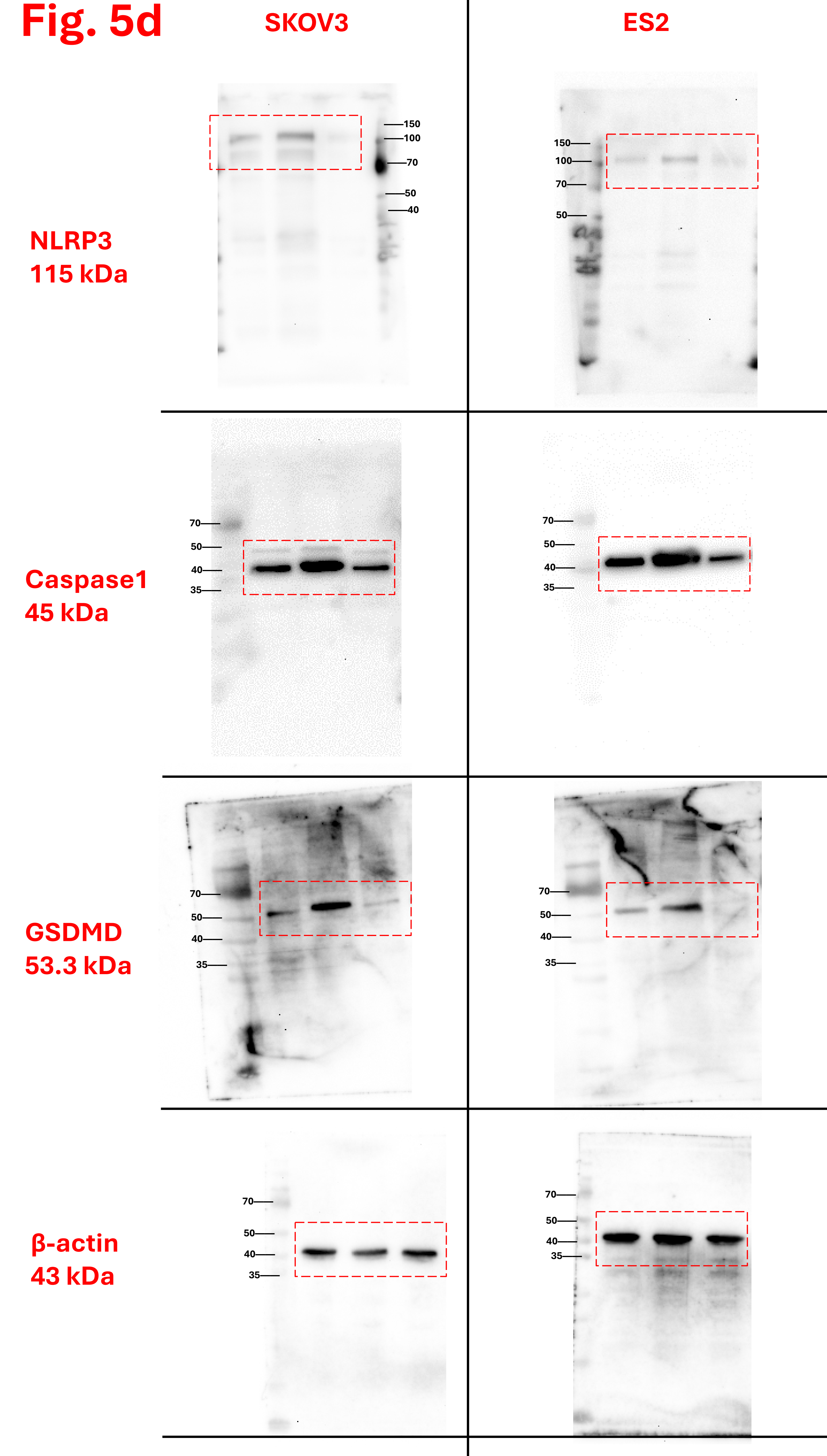
SKOV3
ES2
NLRP3
115 kDa
150
100
70
50
40
Caspase1
45 kDa
70
50
40
35
GSDMD
53.3 kDa
70
50
40
35
β-actin
43 kDa
70
50
40
35

Fig. 7c

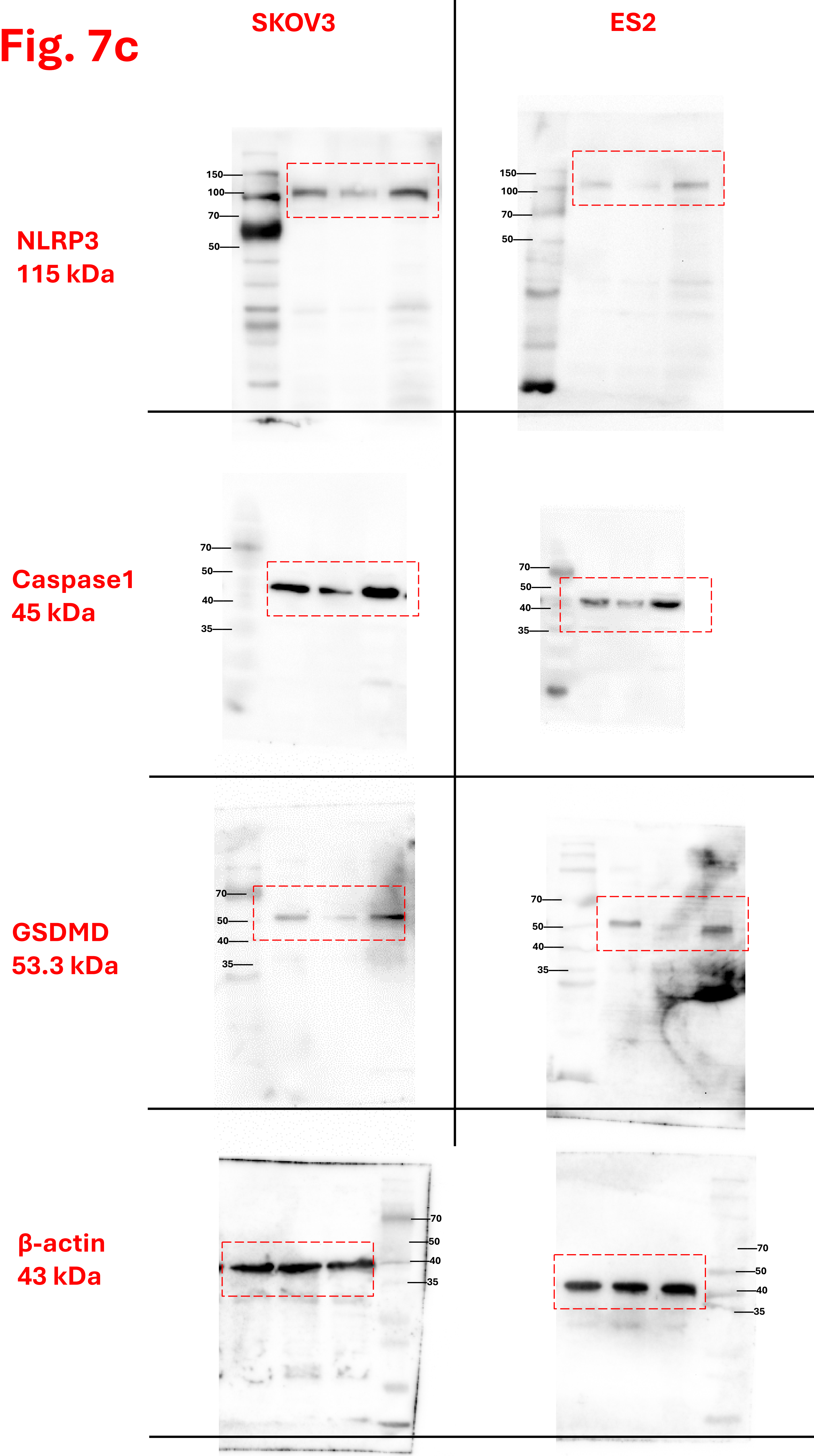

SKOV3
ES2
NLRP3
115 kDa
Caspase1
45 kDa
GSDMD
53.3 kDa
β-actin
43 kDa
150
100
70
50
40
35

Fig. 7d

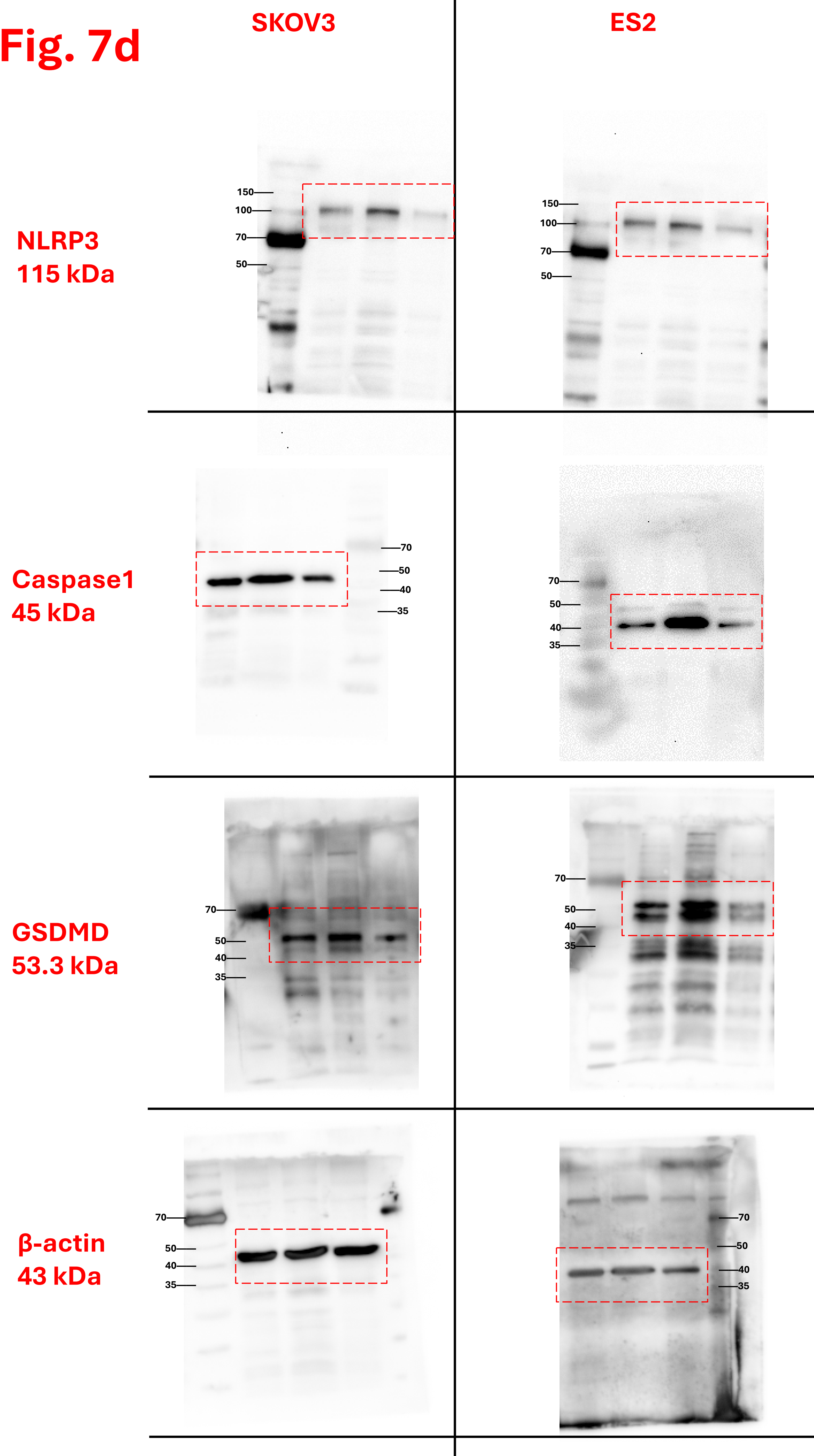

SKOV3
ES2
NLRP3
115 kDa
Caspase1
45 kDa
GSDMD
53.3 kDa
β-actin
43 kDa
150
100
70
50
40
35

Fig. 7e

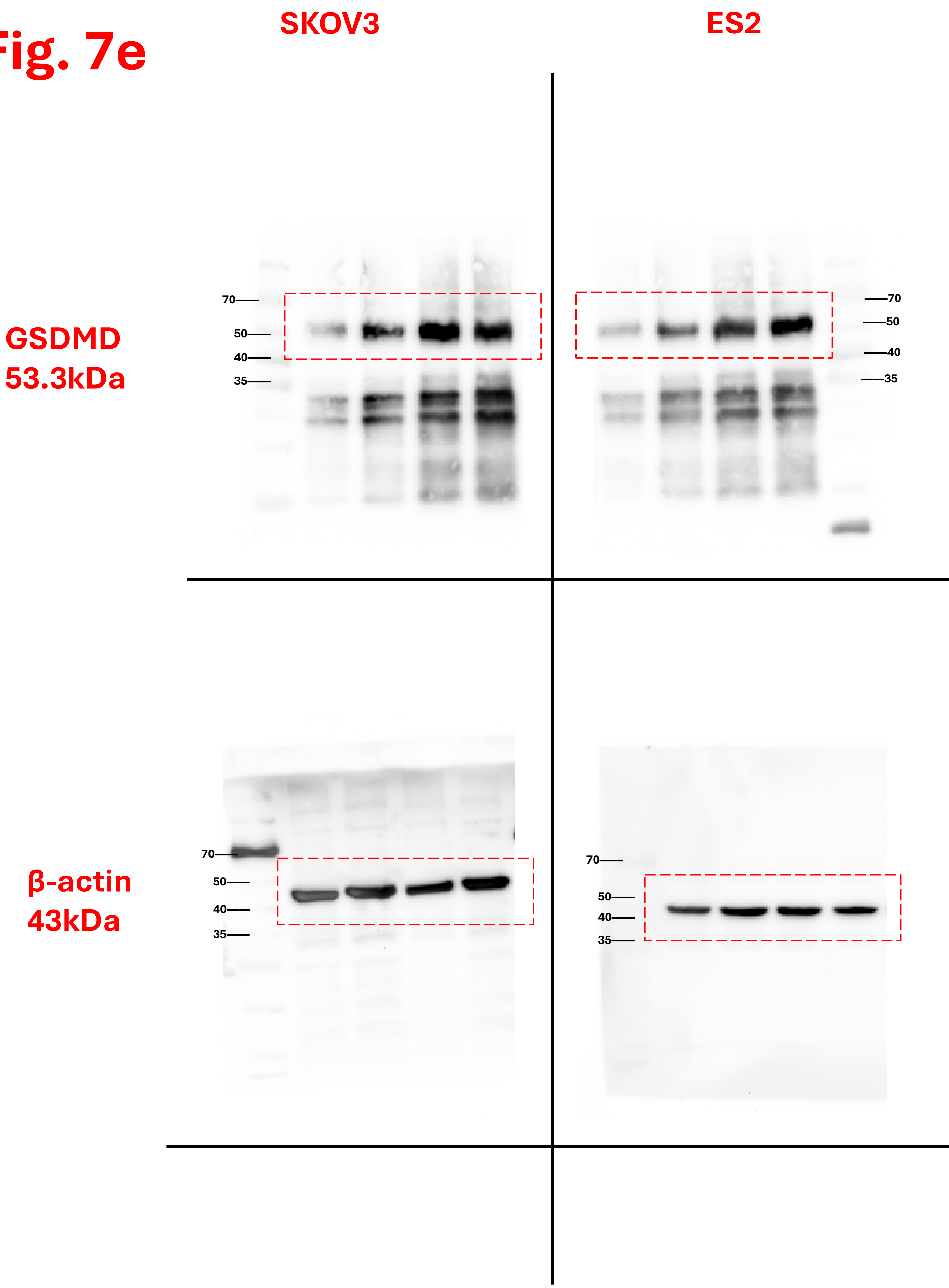
SKOV3
ES2
GSDMD
53.3kDa
β-actin
43kDa
70
50
40
35
70
50
40
35
70
50
40
35
70
50
40
35

Fig. 7f

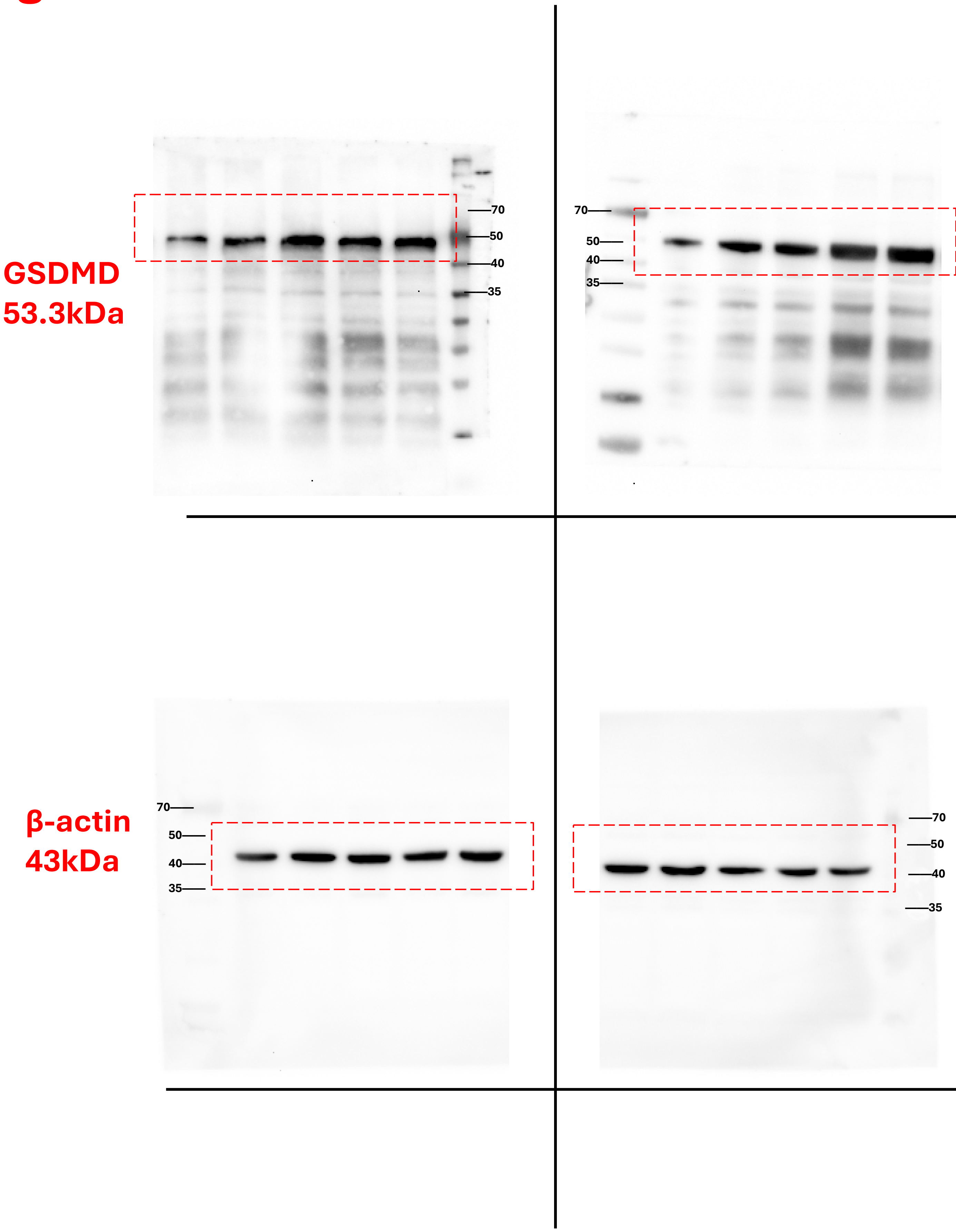

Fig. 7g

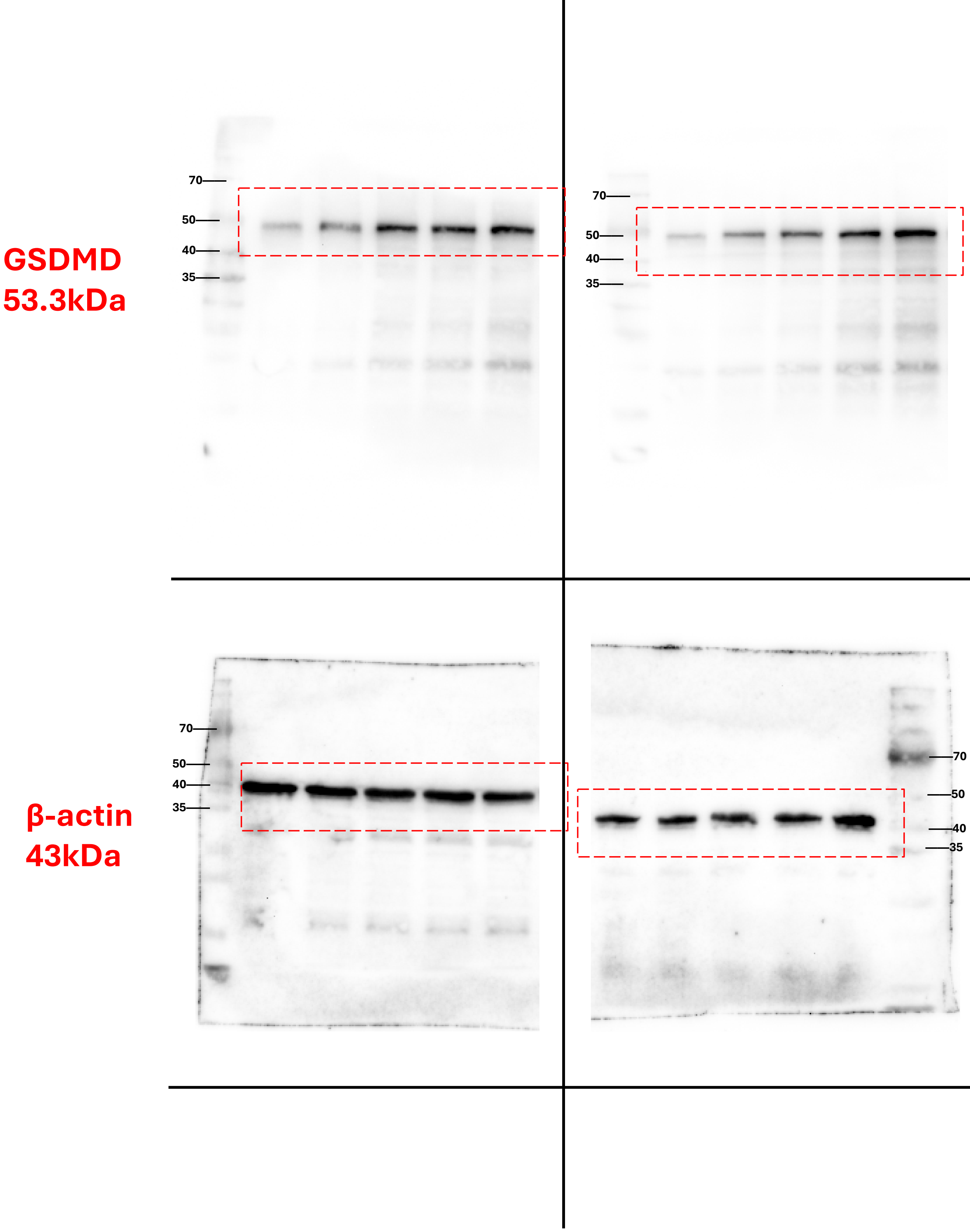
SKOV3
ES2
GSDMD
53.3kDa
β-actin
43kDa
70
50
40
35
70
50
40
35
70
50
40
35
70
50
40
35

Fig. 7h

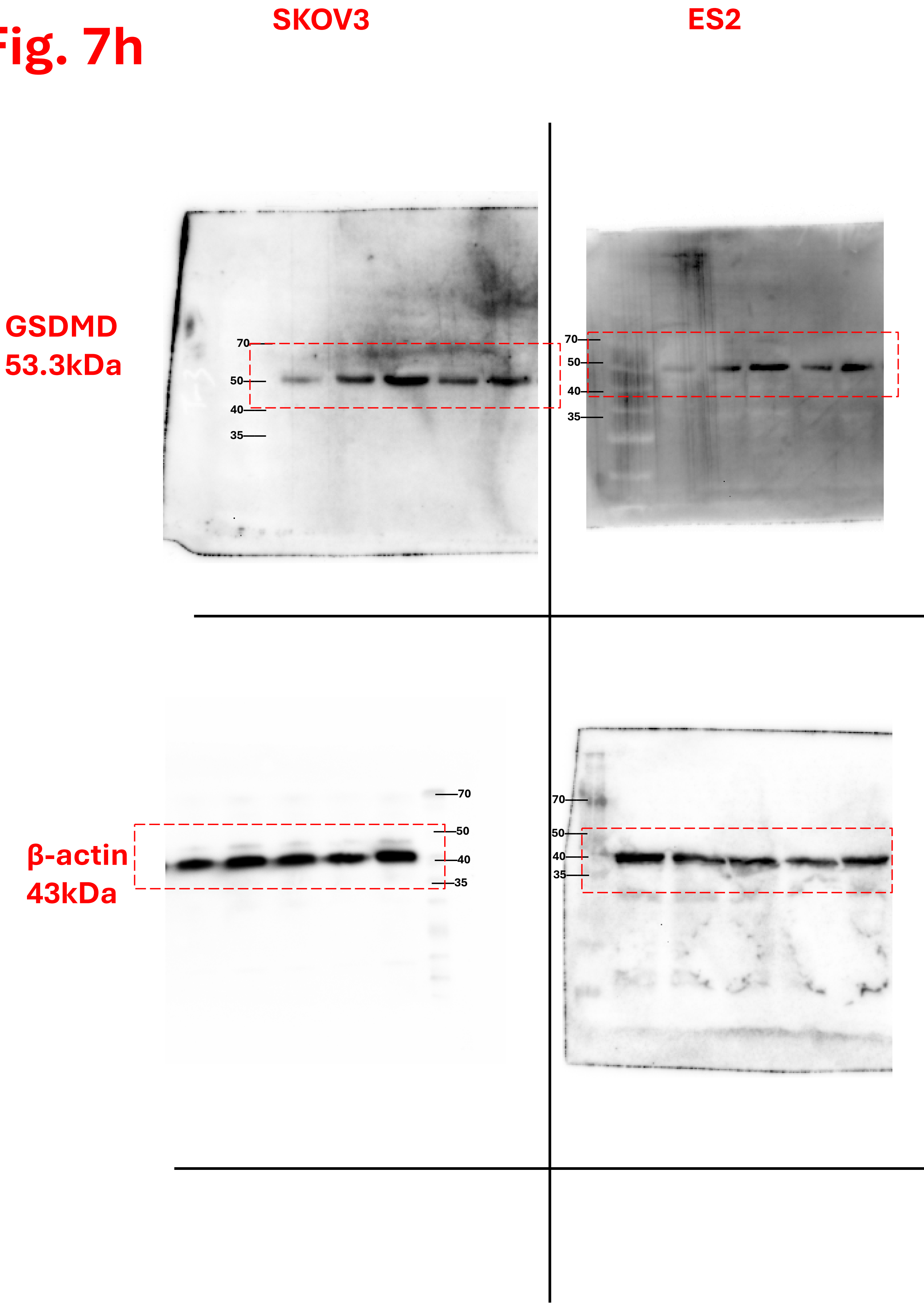

Fig. 5b

SKOV3 | ES2

GSDMD-N
32kDa

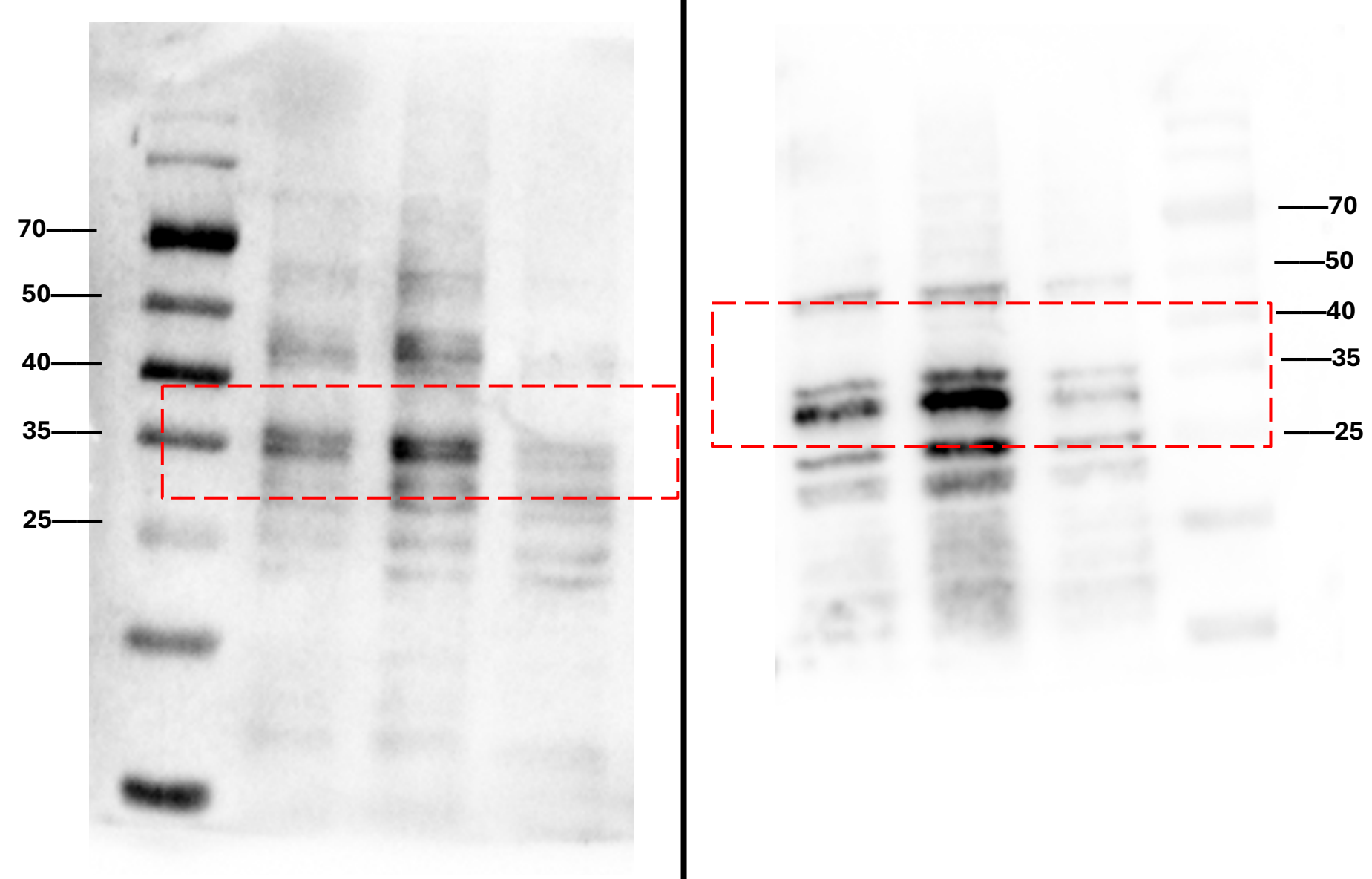

Fig. 5c

GSDMD-N
32kDa

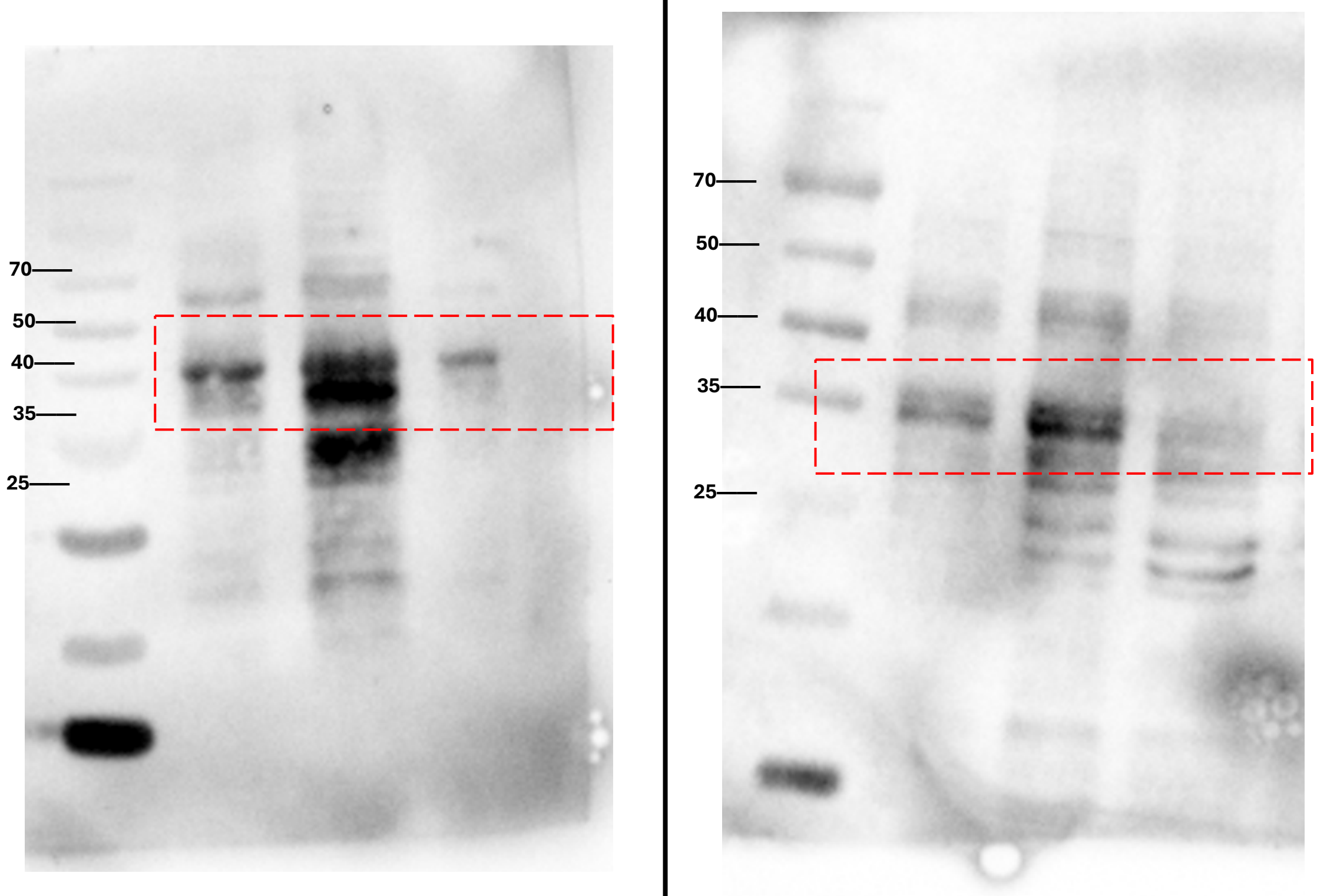

Fig. 5d

GSDMD-N
32kDa

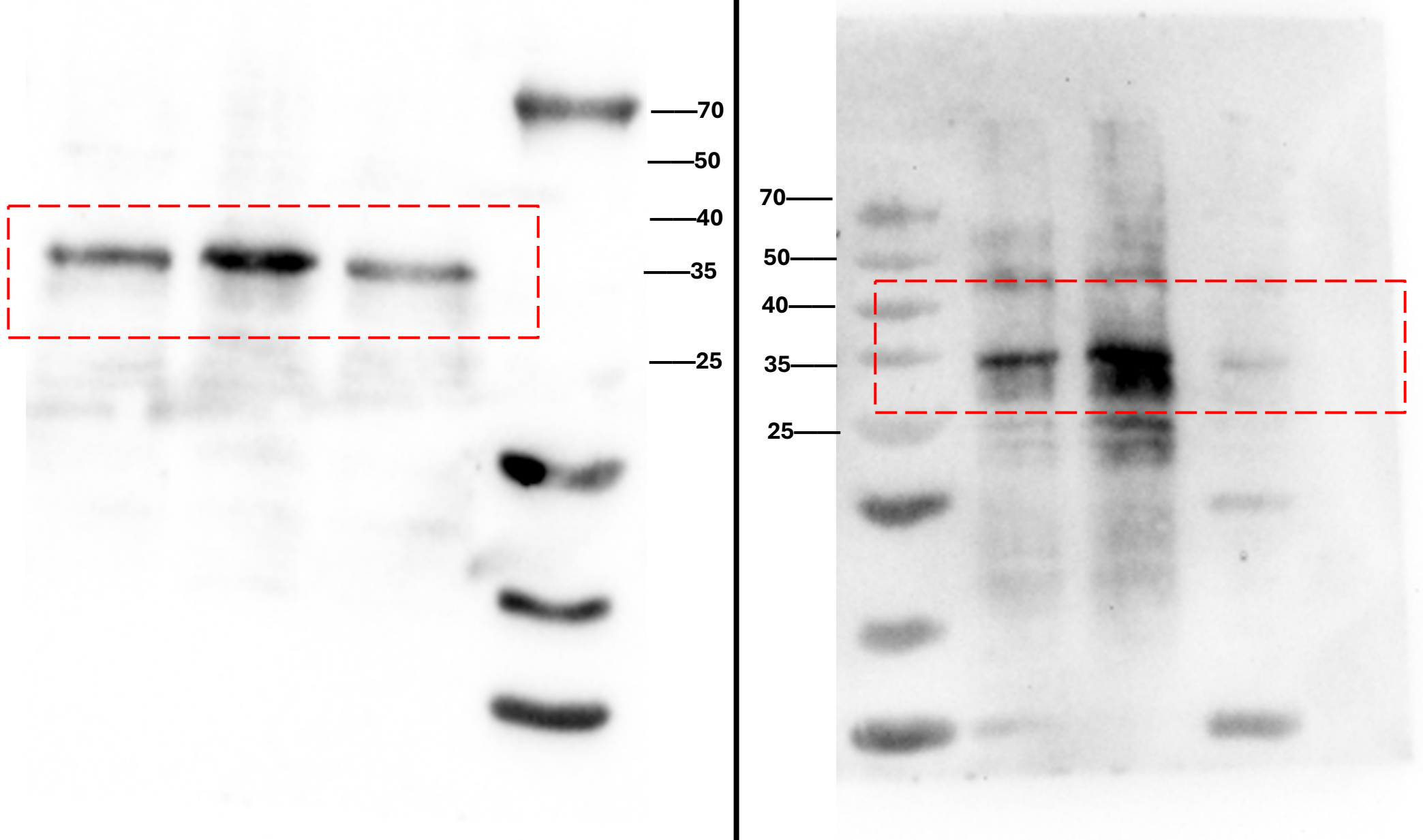

Fig. 7c

SKOV3 **ES2**

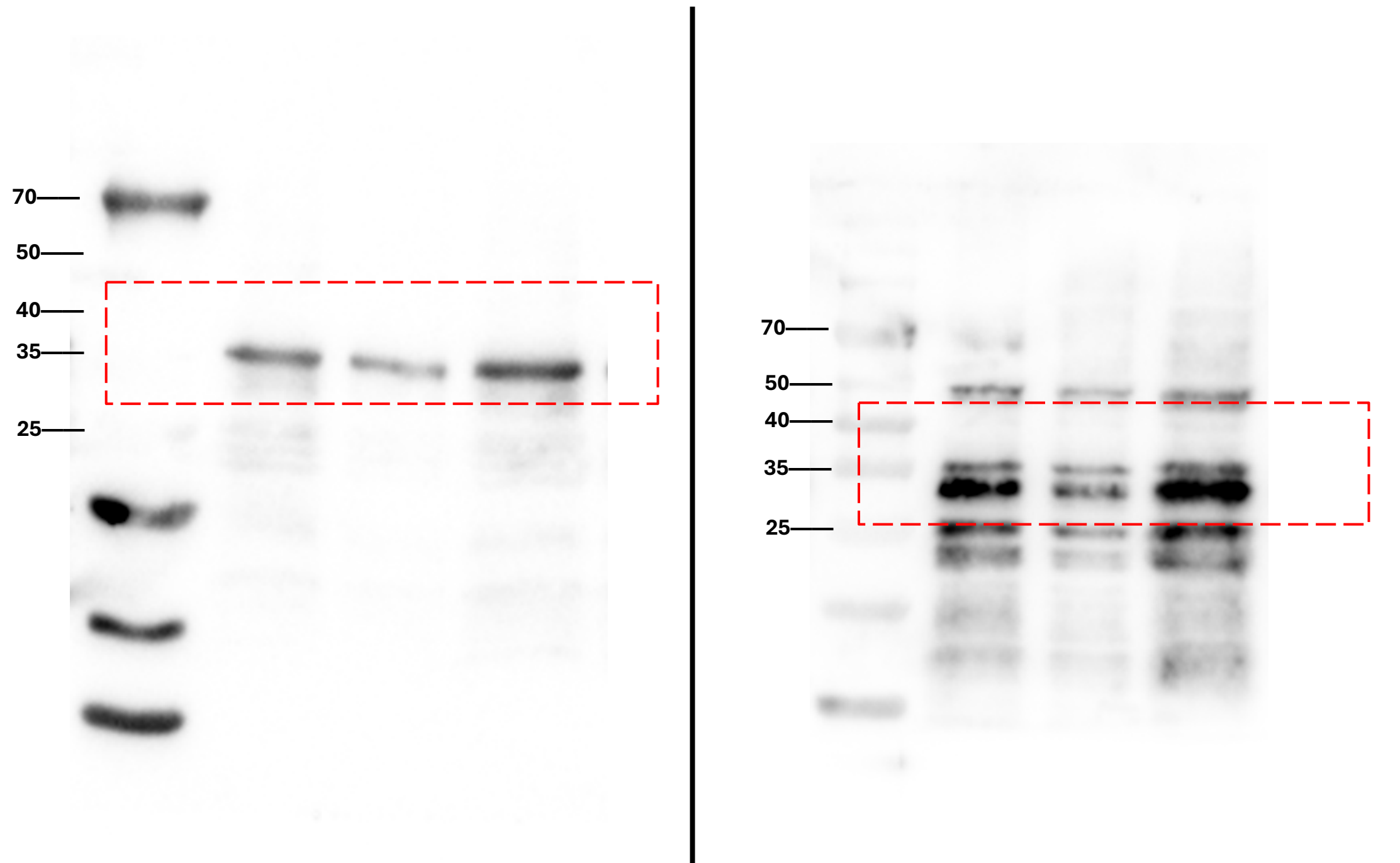

GSDMD-N 32kDa

Fig. 7d

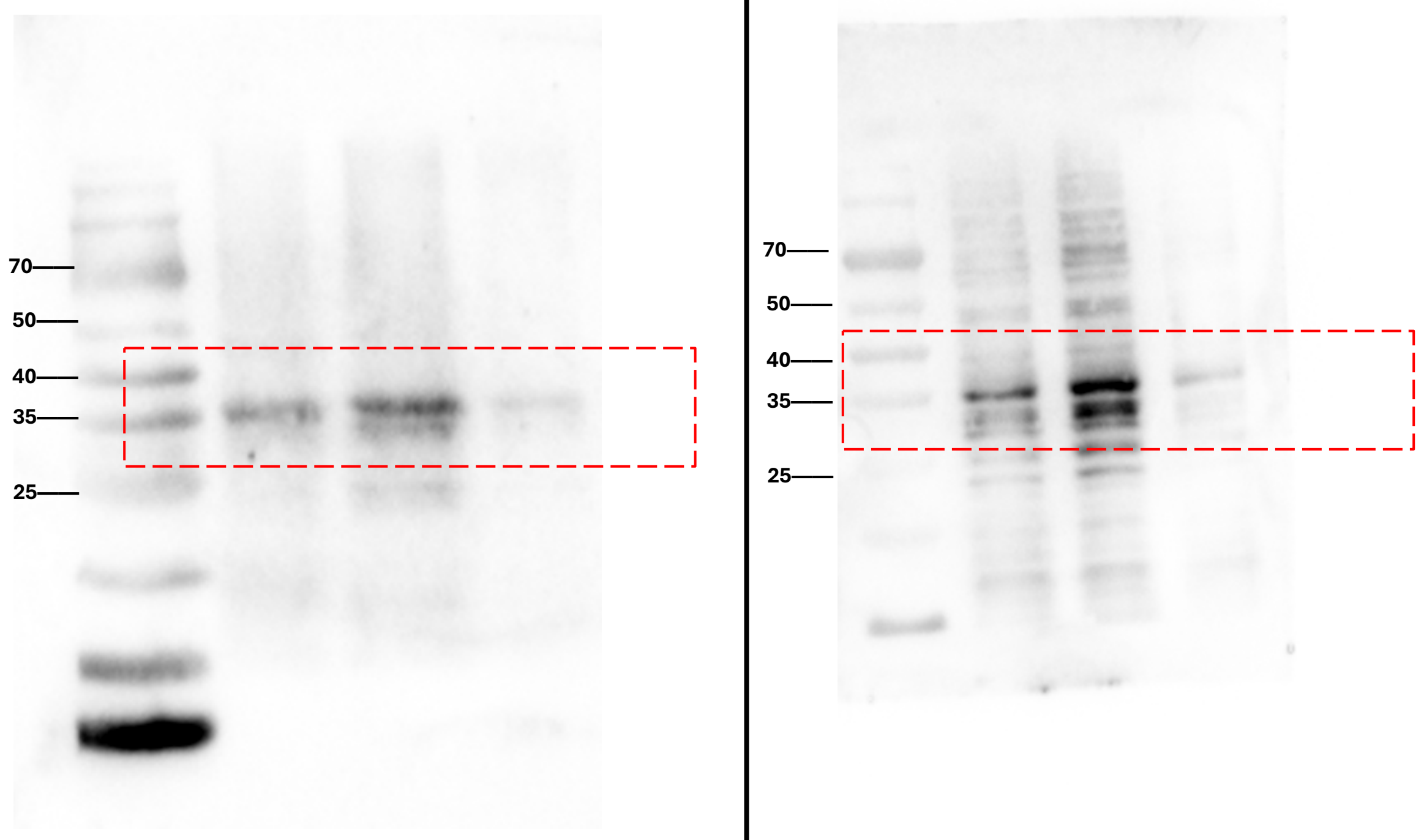

GSDMD-N 32kDa

Fig. 7e

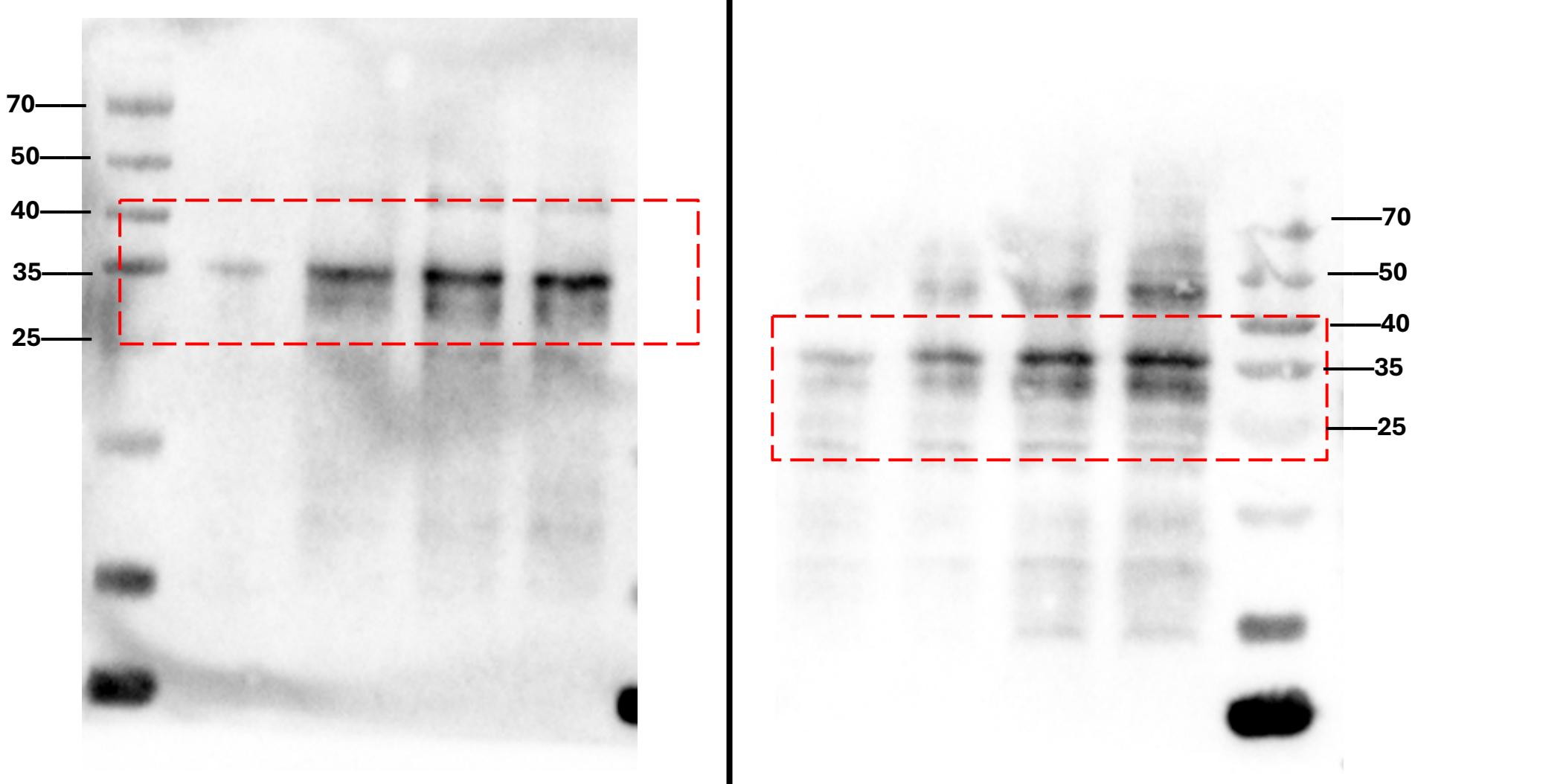

GSDMD-N 32kDa

SKOV3

ES2

Fig. 7f

GSDMD-N
32kDa

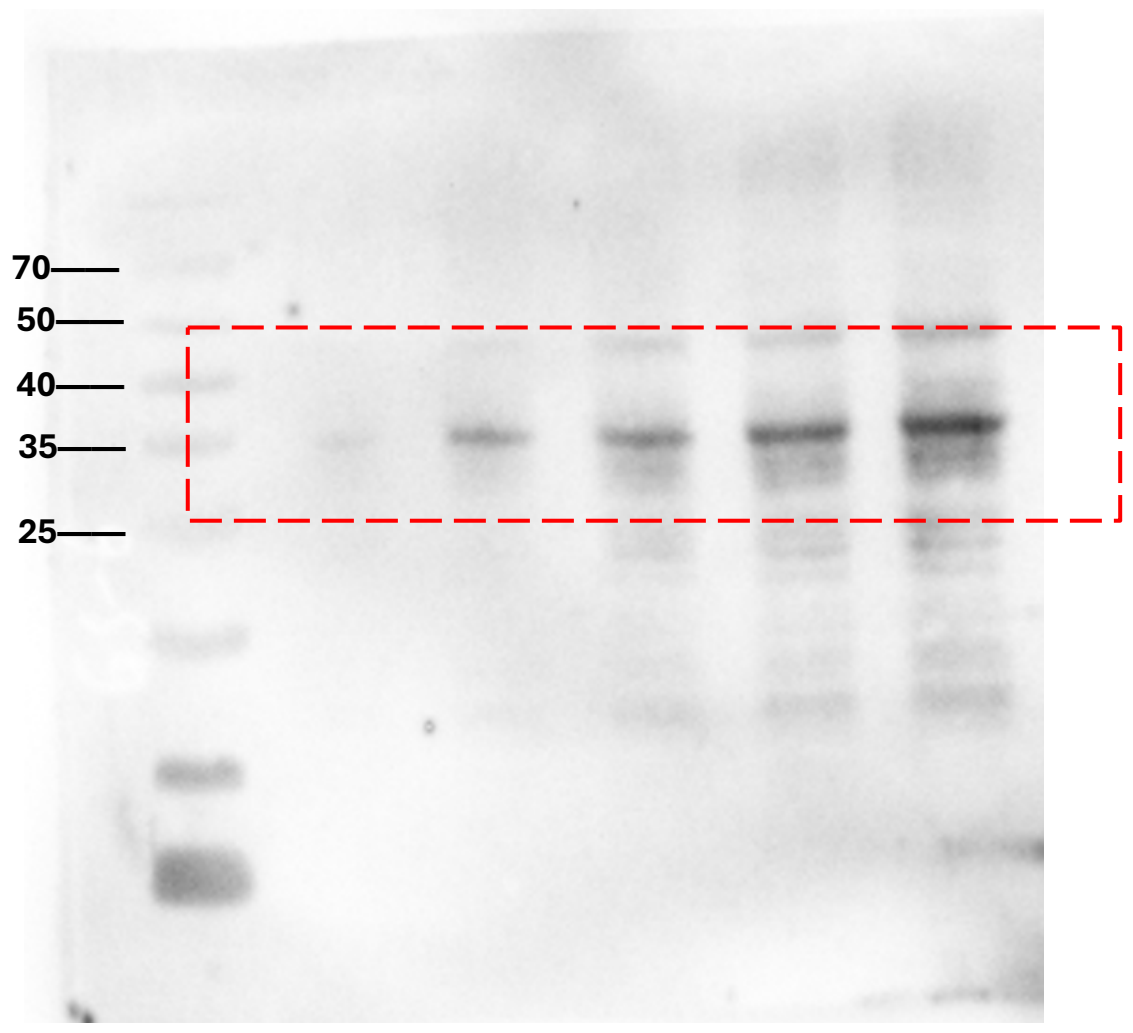

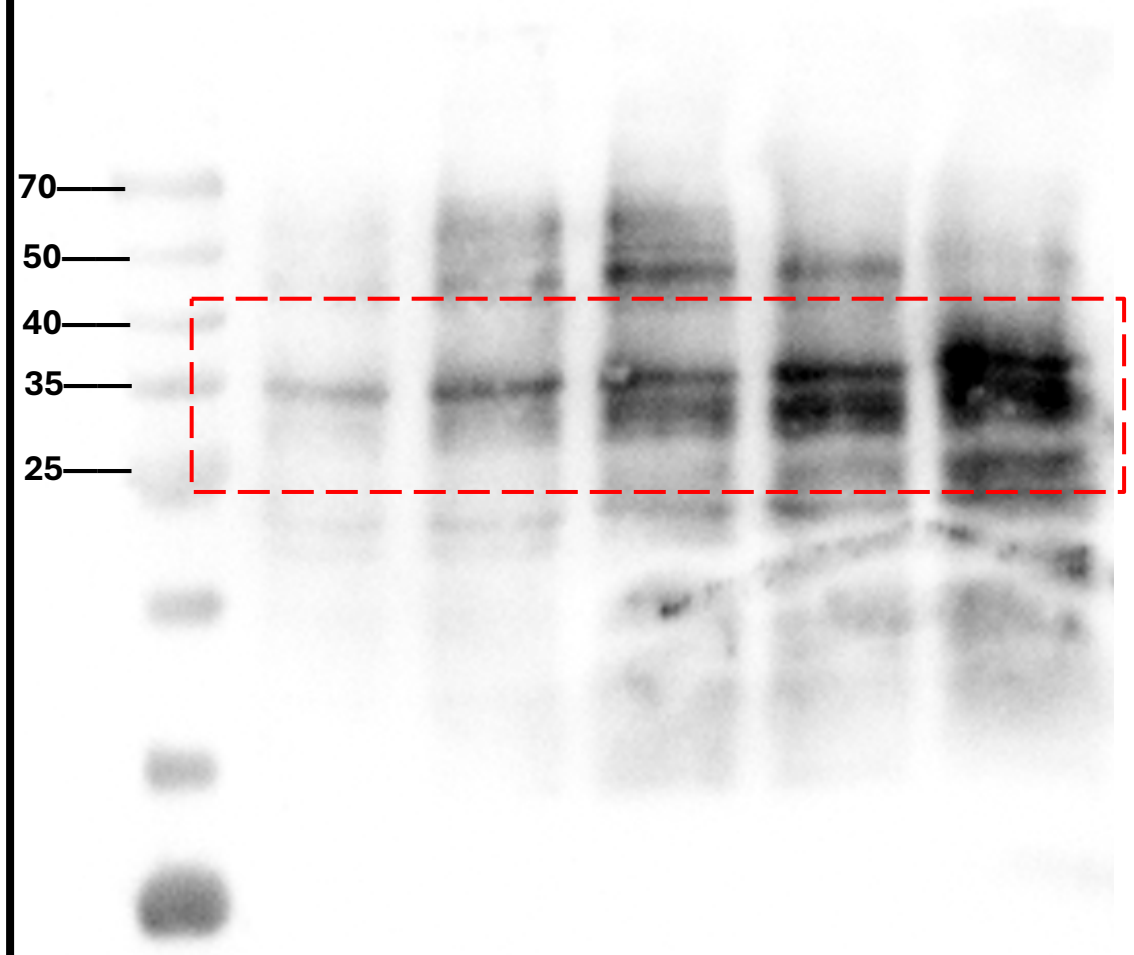

Fig. 7g

GSDMD-N
32kDa

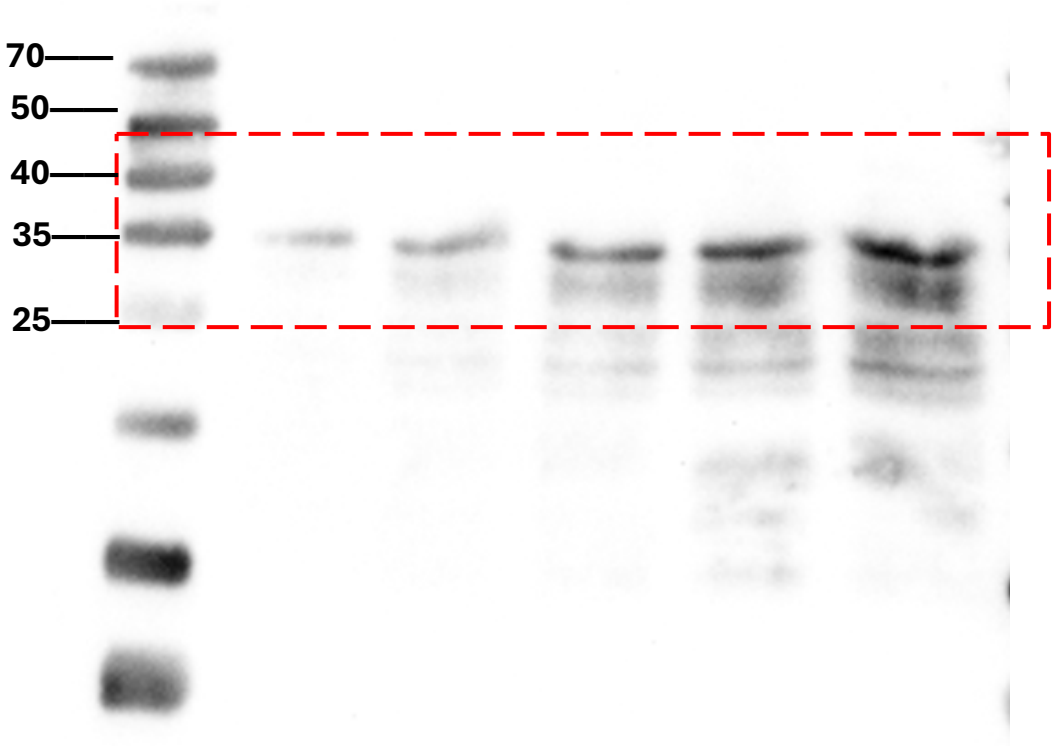

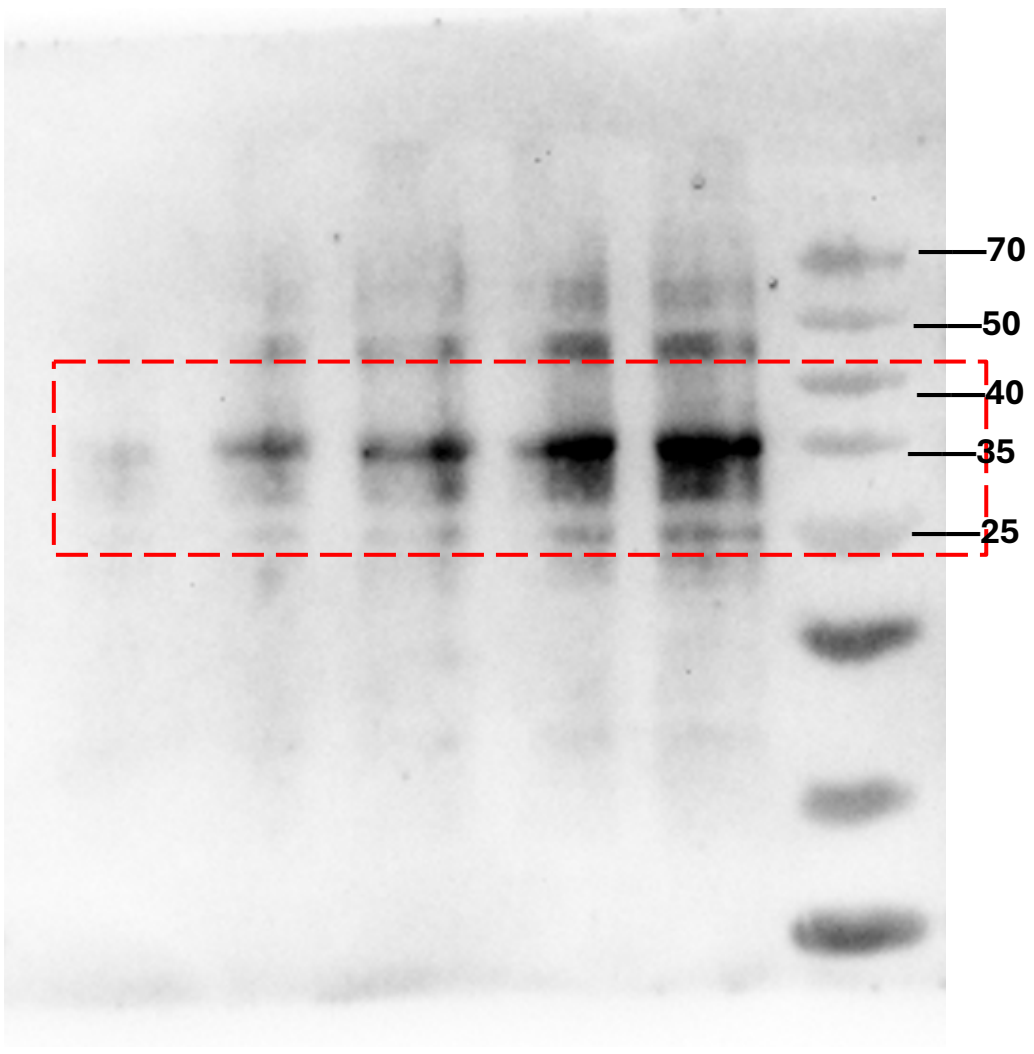

Fig. 7h

GSDMD-N
32kDa

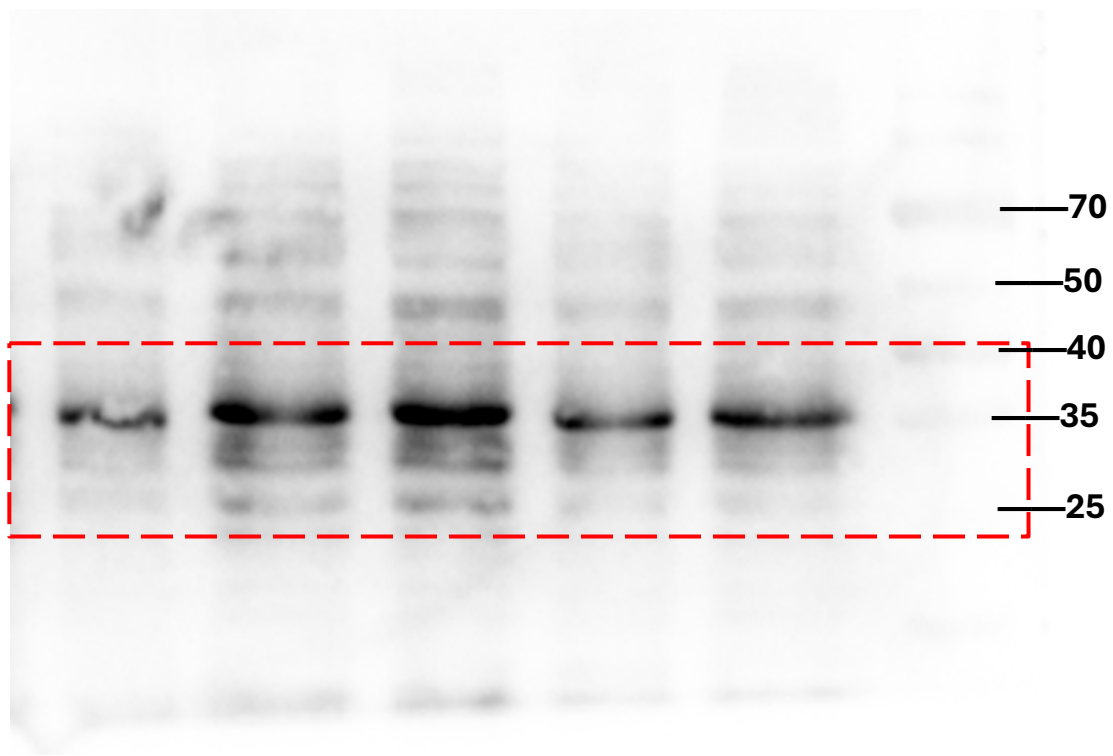

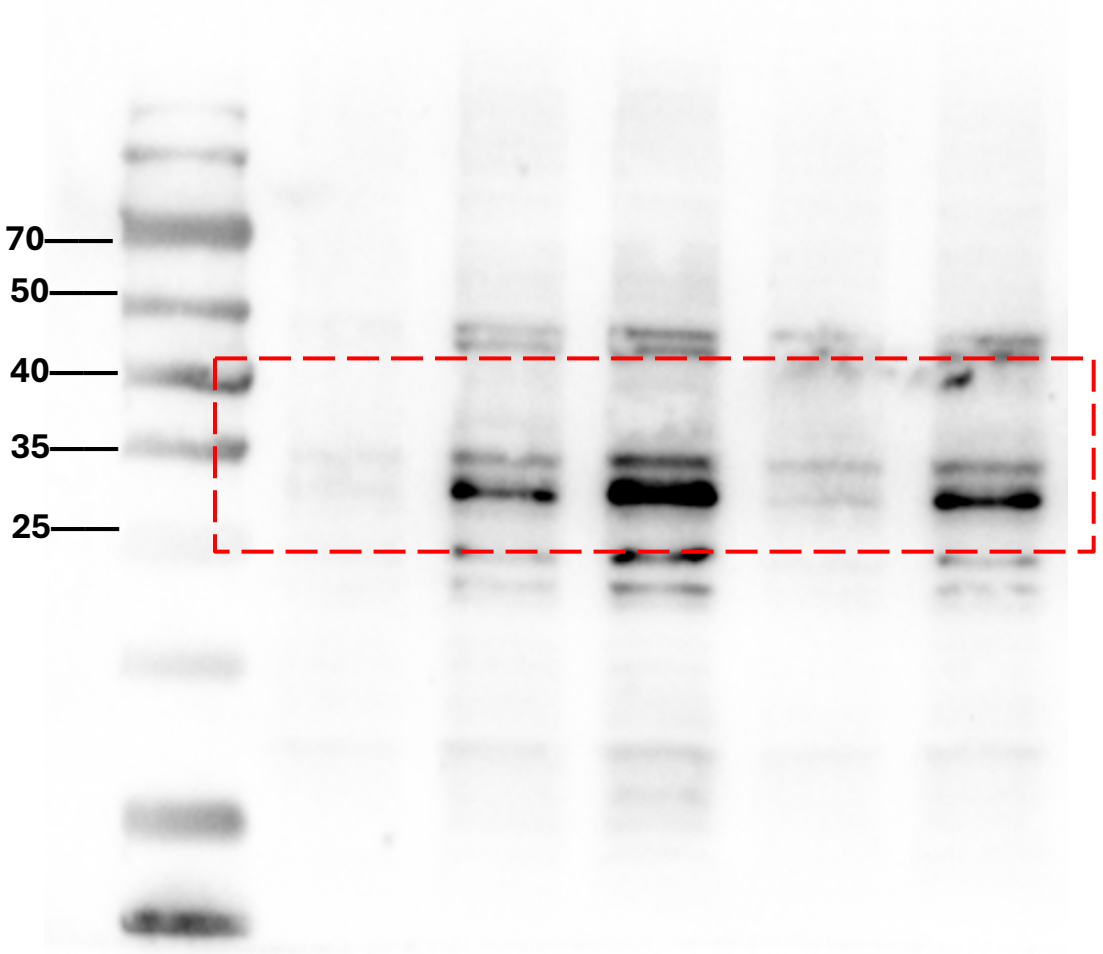

Supplement: Supplementary file 2 [file 41419_2026_8986_MOESM3_ESM.pdf]
