# Supplementary material for: Metabolic activation of LDHA by ERRα represses inflammasome-dependent pyroptosis and promotes ovarian cancer chemoresistance
Source: Cell Death Dis. 2026 Jun 17;17(1):754. doi: 10.1038/s41419-026-08986-6 (PMC13507210; doi:10.1038/s41419-026-08986-6)
Supplement: Supplementary file 3 [file 41419_2026_8986_MOESM1_ESM.docx]

|  | **Overall**  **(n=105)** | **Chemosensitivity**  **(n=76)** | **Chemoresistance**  **(n=29)** | **p-value** |
| --- | --- | --- | --- | --- |
| **Age, years**  (median [IQR]) | 48.00  [40.75, 58.00] | 48.00  [43.50, 58.00] | 51.00  [36.00, 58.00] | 0.64 |
| **BMI, kg/m^2^**  (median [IQR]) | 22.45  [20.92, 25.03] | 23.11  [21.00, 25.70] | 21.80  [20.80, 24.20] | 0.11 |
| **Menopause** (n, %) |  |  |  | 0.503 |
| No | 61 (58.7) | 46 (61.3) | 15 (51.7) |  |
| Yes | 43 (41.3) | 29 (38.7) | 14 (48.3) |  |
| **FIGO stage** (n, %) |  |  |  | 0.989 |
| I | 38 (40.4) | 28 (40.6) | 10 (40.0) |  |
| II | 13 (13.8) | 10 (14.5) | 3 (12.0) |  |
| III | 36 (38.3) | 26 (37.7) | 10 (40.0) |  |
| IV | 7 (7.4) | 5 (7.2) | 2 (8.0) |  |
| **Glucose**  (median [IQR]) | 5.07  [4.59, 5.59] | 5.14  [4.62, 5.65] | 5.05  [4.57, 5.40] | 0.33 |
| **LDH**  (median [IQR]) | 203.40  [166.40, 267.80] | 202.50  [173.68, 268.03] | 219.60  [157.40, 246.10] | 0.641 |

**Table S1 Clinical characteristics of ovarian cancer based on Carb+PTX resistance status**

**Notes:** For continuous nonnormal variables, it showed median and IQR, and p-value was from nonparametric tests. For categorical variables, it showed the percentage that each variable accounted for. BMI, body mass index. LDH, Lactate Dehydrogenase.

**Table S2 Clinical characteristics of ovarian cancer based on DDP+PTX resistance status**

|  | **Overall**  **(n=105)** | **Chemosensitivity**  **(n=84)** | **Chemoresistance**  **(n=21)** | **p-value** |
| --- | --- | --- | --- | --- |
| **Age, years**  (median [IQR]) | 48.00  [40.75, 58.00] | 49.00  [43.50, 58.50] | 45.00  [36.00, 56.00] | 0.288 |
| **BMI, kg/m^2^**  (median [IQR]) | 22.45  [20.92, 25.03] | 23.10  [20.75, 25.50] | 22.39  [21.49, 23.58] | 0.704 |
| **Menopause** (n, %) |  |  |  | 0.503 |
| No | 61 (58.7) | 49 (59.0) | 12 (57.1) |  |
| Yes | 43 (41.3) | 34 (41.0) | 9 (42.9) |  |
| **FIGO stage** (n, %) |  |  |  | 0.446 |
| I | 38 (40.4) | 33 (44.0) | 5 (26.3) |  |
| II | 13 (13.8) | 10 (13.3) | 3 (15.8) |  |
| III | 36 (38.3) | 26 (34.7) | 10 (52.6) |  |
| IV | 7 (7.4) | 6 (8.0) | 1 (5.3) |  |
| **Glucose**  (median [IQR]) | 5.07  [4.59, 5.59] | 5.07  [4.62, 5.54] | 5.17  [4.57, 5.62] | 0.981 |
| **LDH**  (median [IQR]) | 203.40  [166.40, 267.80] | 202.50  [166.38, 268.03] | 226.10  [171.90, 261.80] | 0.741 |

**Notes:** For continuous nonnormal variables, it showed median and IQR, and p-value was from nonparametric tests. For categorical variables, it showed the percentage that each variable accounted for. BMI, body mass index. LDH, Lactate Dehydrogenase.
